# Adult phenotypes of genetic developmental and epileptic encephalopathies

**DOI:** 10.1093/braincomms/fcaf028

**Published:** 2025-01-20

**Authors:** Angeliki Vakrinou, Susanna Pagni, James D Mills, Lisa M Clayton, Simona Balestrini, Sanjay M Sisodiya

**Affiliations:** Department of Clinical and Experimental Epilepsy, UCL Queen Square Institute of Neurology, London WC1N 3BG, UK; Chalfont Centre for Epilepsy, Bucks SL9 0RJ, UK; Department of Clinical and Experimental Epilepsy, UCL Queen Square Institute of Neurology, London WC1N 3BG, UK; Chalfont Centre for Epilepsy, Bucks SL9 0RJ, UK; Department of Clinical and Experimental Epilepsy, UCL Queen Square Institute of Neurology, London WC1N 3BG, UK; Chalfont Centre for Epilepsy, Bucks SL9 0RJ, UK; Department of (Neuro)Pathology, Amsterdam Neuroscience, Amsterdam UMC, University of Amsterdam, Amsterdam 1105 AZ, The Netherlands; Department of Clinical and Experimental Epilepsy, UCL Queen Square Institute of Neurology, London WC1N 3BG, UK; Chalfont Centre for Epilepsy, Bucks SL9 0RJ, UK; Department of Clinical and Experimental Epilepsy, UCL Queen Square Institute of Neurology, London WC1N 3BG, UK; Chalfont Centre for Epilepsy, Bucks SL9 0RJ, UK; Neuroscience and Medical Genetics, Department, Meyer Children’s Hospital IRCSS-University of Florence, Viale Pieraccini 24, 50139 Firenze, Italy; Department of Clinical and Experimental Epilepsy, UCL Queen Square Institute of Neurology, London WC1N 3BG, UK; Chalfont Centre for Epilepsy, Bucks SL9 0RJ, UK

**Keywords:** developmental and epileptic encephalopathies, epileptic encephalopathy, adulthood, EEG, gene expression

## Abstract

Developmental and epileptic encephalopathies constitute a group of severe epilepsies, with seizure onset typically occurring in infancy or childhood, and diverse clinical manifestations, including neurodevelopmental deficits and multimorbidities. Many have genetic aetiologies, identified in up to 50% of individuals. Whilst classically considered paediatric disorders, most are compatible with survival into adulthood, but their adult phenotypes remain inadequately understood. This cross-sectional study presents detailed phenotypes of 129 adults (age range 17–71 years), with genetic developmental and epileptic encephalopathies involving causal variants in 42 genes. We describe diverse disease aspects, and we sought genetic insights from the age-related trends of expression of the genes involved. Most developmental and epileptic encephalopathies (69.7%) are epileptic encephalopathies in adulthood, with the presence of epileptic encephalopathy correlating with worse cognitive phenotypes (*P* = 0.0007). However, phenotypic variability was observed, ranging from those with epileptic encephalopathy to seizure-free individuals with normal EEG or intermediate clinical and EEG phenotypes. This variability was found across individual genes and age-related gene expression trends, suggesting that other influential factors are likely at play. Mobility, feeding and communication impairments were common, with significant dependence on others for activities of daily living. Neurological and psychiatric comorbidities were most prevalent, along with additional systemic comorbidities observed, particularly musculoskeletal, cardiac and gastrointestinal conditions, highlighting the need for comprehensive and multisystemic monitoring. Despite an average diagnostic delay of 25.2 years, aetiology-based therapeutic interventions were feasible for 54.8% of the cohort, underscoring the critical need for genome-wide genetic testing for adults with these phenotypes. Optimizing seizure control remains necessary, but it may not be sufficient to ensure good outcomes, which may differ significantly from childhood metrics, like cognitive function and independence in daily living. Therapies addressing additional aspects beyond seizures are necessary for improving overall outcomes. Understanding the intricate relationship between molecular pathways and the age-related trends of gene expression is crucial for development of appropriate gene-specific therapies and timely intervention. Whilst prospective data are also needed to define these complexities, such studies of necessity take years to acquire: insights from adults can inform care strategies for both paediatric and adult populations now.

## Teaser

Vakrinou *et al.* conducted a cross-sectional study on adults with genetic developmental and epileptic encephalopathies, revealing diverse clinical, EEG, and comorbidity phenotypes across different gene expression trends. Findings suggest influences beyond causal mutations alone, underscoring the need for targeted interventions and comprehensive adult care beyond seizure control.

## Graphical Abstract

**Graphical Abstract:**
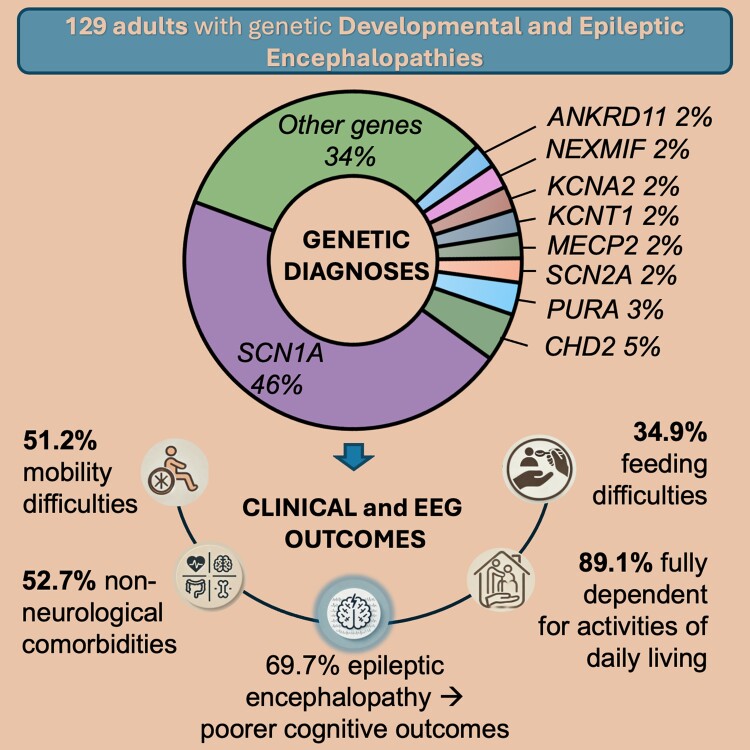

## Introduction

Developmental and epileptic encephalopathies (DEEs) are a group of severe epilepsies, with seizure onset typically occurring in infancy or childhood, usually associated with adverse neurodevelopmental trajectories and multimorbidities. The term DEE encapsulates both a ‘developmental’ component where the developmental deficit is directly linked to the underlying aetiology and an ‘epileptic’ component where seizures and frequent epileptiform activity contribute to developmental slowing or regression.^1^

Genetic aetiologies are common in DEEs, with around 50% currently identified as monogenic disorders.^2^ Over 800 monogenic epilepsy genes have been discovered so far, 90% of which are linked to DEEs.^3,4^ The estimated cumulative incidence of DEEs in children younger than 16 years of age is 169/100 000 or ∼1 in 590.^5^ Given their onset typically in infancy or childhood, genetic DEEs are often considered ‘paediatric’ disorders, to the widespread detriment of both older individuals who risk remaining undiagnosed, and younger individuals and families, for whom longer term outcome data are essential: most published studies on their aetiology, clinical presentation, progression and novel treatments, focus on paediatric cohorts.^6-8^ Only recently have publications described adult populations with genetic DEEs, mainly focusing on the diagnostic yield of next generation sequencing testing in these populations, which in fact is comparable to testing in paediatric populations, ranging from 25 to 50%.^9-11^ Nevertheless, studies concerning adult DEE phenotypes are still limited and primarily take a gene-based approach.^12-18^ Furthermore, as care of children with genetic DEEs improves, we can expect a greater proportion of such children to survive to adulthood. A recent population study estimated that >70% of children with monogenic epilepsy will likely need neurological care into adulthood.^19^ Despite these recent advances and the clear need for more information, data on the phenotypic spectrum of DEEs in adults remain limited.

The multitude and complexity of clinical problems associated with DEEs have a profound impact on the affected individuals and their families.^20^ Moreover, the economic burden is significant, both in the health care setting, given their morbidity and premature mortality,^21^ but also in the community, as most affected individuals will require lifelong care.^22,23^ Therefore, developing therapies to address these complex disorders is imperative. Pioneering gene-based therapies for DEEs are considered a realistic option for the near future.^24^ However, the lack of both comprehensive data on clinical outcomes and guidance on suitable clinical outcome assessment tools, especially in adulthood, for these rare conditions poses significant challenges for clinical trial readiness for novel therapies.^25,26^ Whilst prospectively-acquired data can be invaluable for such purposes, by default it takes years to acquire. By contrast, insights gathered directly during adulthood has the potential to inform prognostication, providing key data for such trials and to optimize genetic counselling for families.^7^

The majority of adults alive today with a genetic DEE will not have benefited from recent diagnostic and treatment discoveries,^27^ such that data on the long-term course could be considered to approximate natural history outcomes, as data are drawn from individuals not intentionally treated with ‘precision’ treatments, or even sometimes inadvertently given inappropriate treatments. We present a series of individuals with genetic DEEs from one specialist centre treating adults with epilepsy. Our study specifically focuses on DEEs with a confirmed causal genetic variant, aiming to provide insight into the adult outcomes of individuals diagnosed with specific genetic DEEs in childhood, rather than DEEs in general. To begin to explore underlying mechanisms, and the value of treatments targeting culpable genes, we also examine the relationship of age-related trends of gene expression with respect to phenotypes of interest.

## Materials and methods

### Ethics approval

This research was approved by the relevant ethics committee (REC 11/LO/2016). Written informed consent for research use of clinical and genetic data was obtained from patients, their parents, or legal guardians for those with intellectual disability (ID).

### Study cohort

We included consecutive adults (≥16 years) attending epilepsy outpatient clinics at the National Hospital for Neurology and Neurosurgery over a 3-year period. Each individual’s case was reviewed by the authors at the time of inclusion, and individuals were selected only if they had met the diagnostic criteria for a DEE, as defined by the 2017 International League Against Epilepsy diagnostic criteria,^1^ at some point in their clinical trajectory. The diagnosis for each individual was established based on comprehensive review of clinical (including neurodevelopmental), EEG, and imaging data, rechecked after genetic findings. Although some individuals may no longer fully meet the criteria for DEE at the time of the study (and documenting this is partly the purpose of the study), their inclusion was based on whether the individuals had met the diagnostic criteria at some point in their clinical course. Causal genetic variants had been classified as pathogenic or likely pathogenic according to American College of Medical Genetics and Genomics guidelines.^28^ Pathogenicity of the identified variants was established following discussion at multidisciplinary meetings (including epileptologists, clinical geneticists and clinical scientists) or after evaluation by an accredited clinical genetics laboratory in the context of an appropriate clinical phenotype according to the International League Against Epilepsy diagnostic criteria.^29^

### Genetic diagnoses

Molecular genetic results were originally obtained through a variety of means including single gene analyses or gene panel sequencing in clinically-accredited laboratories, research whole exome sequencing or whole genome sequencing through the 100 000 Genomes Project.^30^ All research findings had already been confirmed in a clinically accredited laboratory using fresh samples from each individual. For cases in whom the genetic diagnosis was made in the 100 000 Genomes Project, only diagnoses made after discussion at a Genomics Medicine Centre multidisciplinary meeting were included: no data were taken directly from the Genomics England research environment, diagnostic discovery or clinical collaboration routes. Segregation analyses were not possible for the entire cohort but are presented where available.

### Age-related trend of gene expression

Bulk RNA-sequencing expression data were obtained from the Allen Brain Atlas^31^ to investigate the age-related profile of gene expression associated with the genes of interest within the study cohort.

In order to systematically classify genes based on their age-related expression profile and distinguish between those with an increasing, decreasing or neutral expression over time, we applied a linear regression analysis for each gene’s expression values across the temporal axis (age range: 8–9 post-conceptual week to 40+ years). Genes with a positive slope in their regression analysis were considered to have an increasing expression trend with age, whereas genes with a negative slope were considered to have a decreasing expression trend. Genes for which the confidence interval of the slope encompassed zero were categorized as having a neutral expression trend (Supplementary Table 1).

### Clinical phenotyping

Phenotypic information was collected through retrospective review of contemporaneous medical records; data collection was completed in January 2024. Demographic and clinical data, where available, included the following: gender, age at time of recruitment, age at time of genetic diagnosis, cognitive abilities (level of ID, if present) at last follow-up, epilepsy phenotypes including age at seizure onset, seizure types and frequency at last follow-up, presence of autism or behavioural problems, presence and types of comorbidities at last follow-up, antiseizure medication (ASM) exposure/responses over time, data on EEG, mobility, feeding, language and dependency for activities of daily living (ADLs) at last follow-up. Seizure frequency, including the presence of drug-resistance or seizure freedom, were determined according to International League Against Epilepsy definitions.^32,33^ We defined ‘frequent seizures’ as those occurring monthly or more frequently, and ‘infrequent seizures’ as those occurring less than monthly. Cognitive abilities were defined based on formal neuropsychometric data, where available, or based on the level of functioning as documented in the medical records from families, carers and treating clinicians.

Encephalopathy on EEG was defined as the presence of slowing of background rhythms (background slowing) and loss of rhythm reactivity.^34,35^ Patients were classified as having epileptic encephalopathy (EE) if they met specific clinical and EEG criteria:

1. Clinical criteria: Presence of seizures within the last year AND concurrent presence of ID and/or cognitive decline.

AND

2. Presence of encephalopathy on EEG.^34,35^

AND

3. Interictal epileptiform abnormalities (focal/multifocal or generalized sharp or spike-slow waves not clearly related to seizures).^35-37^

OR

Ictal epileptiform abnormalities (focal, multifocal or generalized sharp and/or (poly)spikes-slow waves and/or episodic fast activity).^35-37^

Although this study primarily focused on phenotypes at last follow-up, we also examined the phenotypes of interest over time through retrospective review of early (paediatric) medical records, where available, to explore disease progression. Such records had not been collected in a systematic way.

### Statistical analysis

Mean and standard deviations (SDs) are given where data are normally distributed, and median and interquartile ranges (IQRs) otherwise. The Mann–Whitney U-test was used to evaluate the association between seizure-onset age and cognitive and functional phenotypes (borderline/mild versus moderate/severe ID; verbal versus non-verbal; ambulant versus non-ambulant; dependent versus independent with ADLs). The χ^2^ test was used to compare (i) seizure phenotypes (frequent versus infrequent/seizure free) or (ii) combined clinical and EEG phenotypes (EE: yes versus no), with cognitive and functional phenotypes (as described above). The χ^2^ test was also used to compare (i) clinical and EEG phenotypes (EE: yes versus no) or (ii) cognitive phenotypes (borderline/mild versus moderate/severe ID) and the different gene expression trends (increasing; decreasing; neutral). Fisher’s exact test was used in case any of the cells had an expected count below 5. Alpha level of significance, following Bonferroni correction for multiple comparisons, was set at *P* < 0.003 (0.05/14 tests). Statistical analyses and figure generation were conducted in the ‘R’ environment.^38,39^

## Results

### Study cohort and genetic diagnoses

**Figure 1:**
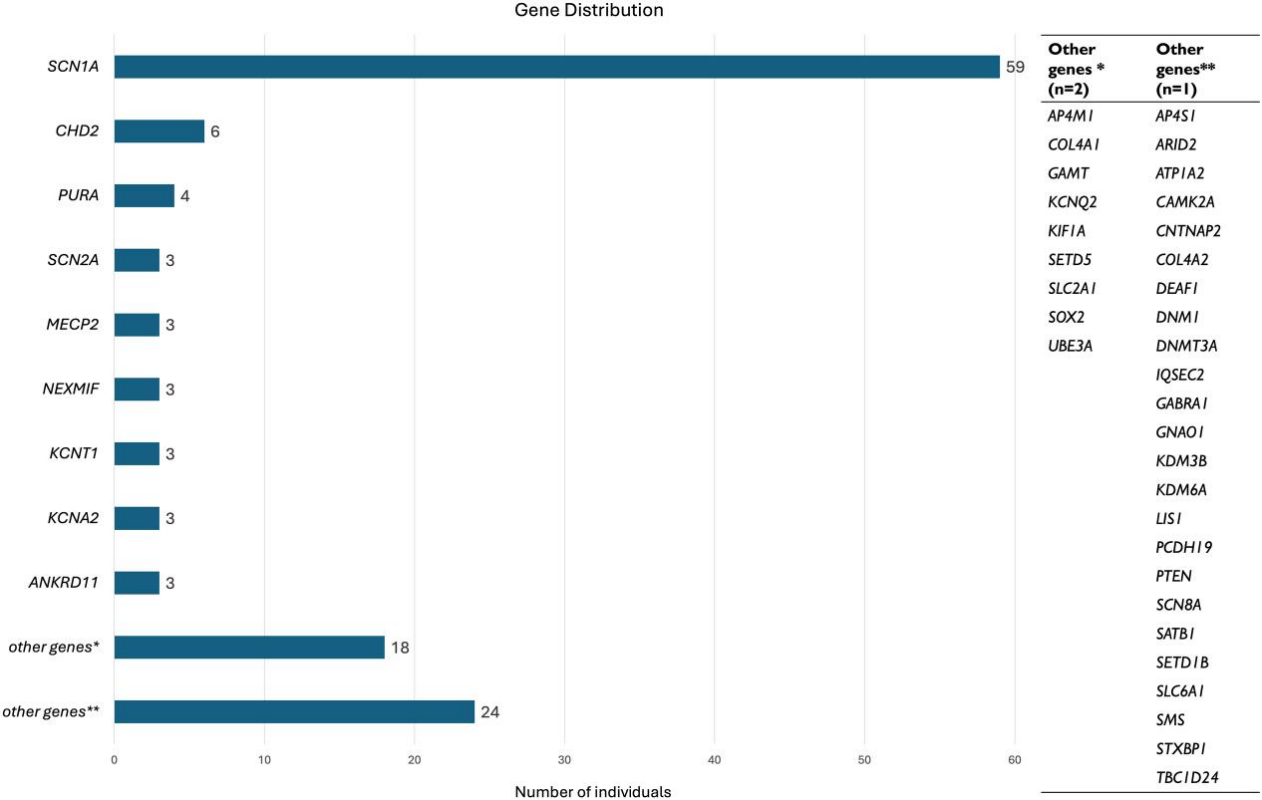
**Gene distribution across the cohort.** The cohort was highly skewed towards *SCN1A*-related DEEs (59/129, 45.7%). Genetic diagnoses involved 42 genes, for the majority of which (33/42 genes, 78.6%), there were single cases (other genes**) or cases with two individuals (other genes*). These 33 genes are listed on the right side of the figure.

A total of 129 adults with a genetic DEE were included in the study (73 females, 56.6%). The median age at inclusion in the study was 30 years (range: 17–71 years); details on the age at inclusion or the age at DEE diagnosis (if made prior to the inclusion in the study) for each adult can be found in Supplementary Table 2. Median age at time of genetic diagnosis was 26 years (IQR: 21–36 years; range: 5–60 years). A genetic diagnosis was established on average 25.2 years (SD: 11.4 years; range: 4–58.5 years) after epilepsy onset. Genetic diagnoses involved 42 genes, the most commonly implicated being *SCN1A* (59/129, 45.7%). In 63/129 (48.8%) individuals, variants occurred *de novo*. Seven individuals (5.4%) carried homozygous variants (with *AP4M1*, *AP4S1*, *CNTNAP2*, *GAMT* and *SCN1A*-related DEEs). In the remaining individuals (59/129; 45.8%), information on variant segregation was unavailable (parental testing not possible) or incomplete (genetic testing only possible in one parent). The distribution of diagnoses by gene is shown in Fig. 1.

The results will be presented by dividing the main cohort into two sub-cohorts: the *SCN1A* cohort (*n* = 59) and the non-*SCN1A* cohort (*n* = 70). In the *SCN1A* cohort, most individuals had Dravet syndrome (55/59; 93.2%), the remaining having other *SCN1A*-related DEEs. A clinical diagnosis of Dravet syndrome was established prior to genetic testing for 41/59 (69.5%) individuals. Clinical syndromic diagnoses were established prior to genetic testing for 5/70 (7.1%) individuals in the non-*SCN1A* cohort: two individuals with Angelman syndrome, and one individual each with Lennox–Gastaut syndrome, Landau–Kleffner syndrome and Jeavons syndrome. The latter three individuals were found to harbour *CHD2* pathogenic variants, two of which were *de novo* (Lennox–Gastaut and Landau–Kleffner syndromes) and one for which inheritance was unknown (Jeavons syndrome).

### Epilepsy phenotypes

By design, all individuals across both cohorts had a diagnosis of epilepsy.

#### *SCN1A* cohort

**Figure 2:**
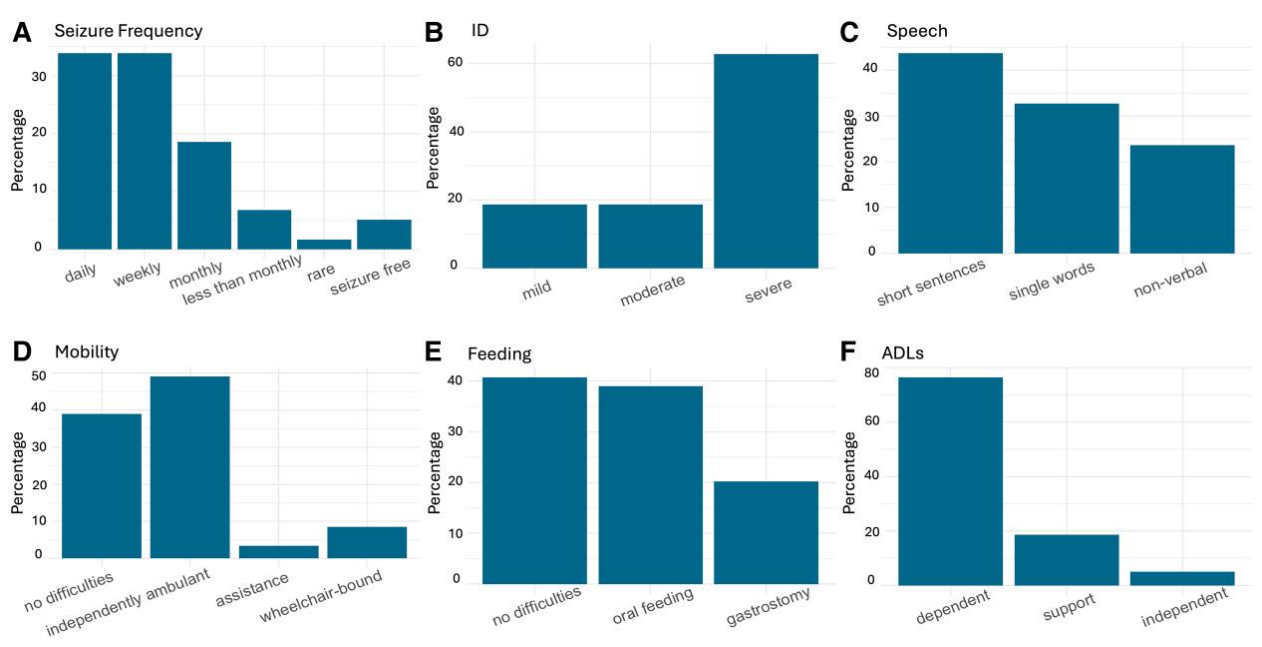
**Clinical outcomes across the *SCN1A* cohort (*n* = 59), all at last follow-up.** (**A**) Prevalence of classes of seizure frequency. (**B**) Level of ID. (**C**) Level of speech impairment. Information on verbal communication was available for 55/59 (93.2%) individuals. (**D**) Mobility outcomes. (**E**) Feeding outcomes. The ‘oral feeding’ group includes individuals with feeding difficulties who were still fed orally. (**F**) Level of independence for ADLs. ID, intellectual disability; ADLs, activities of daily living.

Information on age at seizure onset was available for 58/59 (98.3%) individuals, with a median onset age of 6 months (IQR: 4–9 months), and median epilepsy duration to last follow up of 31.79 years (IQR: 24.5–41.3 years). Three individuals (3/59, 5.1%) were seizure-free at last follow-up, aged 26, 37 and 54 years. The remaining individuals had drug-resistant epilepsy, with 51/59 (86.4%) experiencing frequent seizures and 5/59 (8.5%) experiencing infrequent seizures at last follow-up (Fig. 2).

#### Non-*SCN1A* cohort

**Figure 3:**
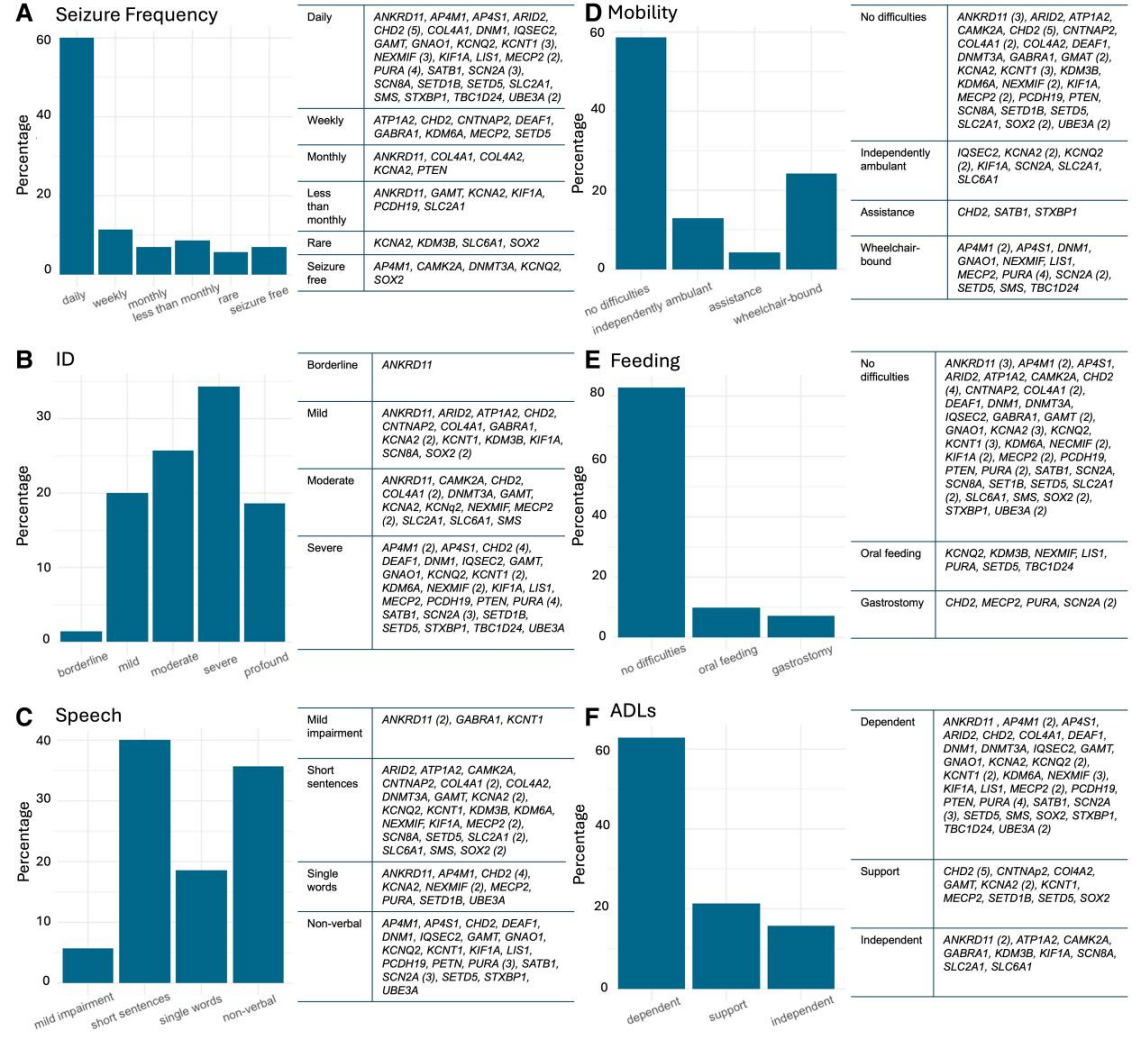
**Clinical outcomes across non-*SCN1A* cohort (*n* = 70) at last follow-up.** Genes implicated in each outcome category are seen on the right side of each figure. (**A**) Prevalence of classes of seizure frequency. (**B**) Level of ID. (**C**) Level of speech impairment. For 4/70 (5.7%) individuals, verbal communication was mildly impaired but no further information was available. (**D**) Mobility outcomes. (**E**) Feeding outcomes. The ‘oral feeding’ group includes individuals with feeding difficulties who were still fed orally. (**F**) Level of independence for ADLs. ID, intellectual disability; ADLs, activities of daily living.

Information on seizure-onset age was available for 66/70 (94.3%) individuals. The median age at seizure onset was 8.5 months (IQR: 5–24 months); median epilepsy duration was 27.8 years (IQR: 21.3–38.4 years). In 25/66 (37.9%), seizures started within the first year of life. Five individuals (5/70, 7.1%) were seizure-free at last follow-up (with *AP4M1*, *CAMK2A*, *DNMT3A*, *KCNQ2* and *SOX2*-related DEEs); their median age was 30.5 years (range: 29–44 years). The remaining 65/70 (92.9%) individuals had drug-resistant epilepsy, with the majority (55/65, 84.6%) experiencing frequent seizures (Fig. 3).

### Clinical and EEG phenotypes and gene expression trends

EEG data in adulthood were available for 99/129 (76.7%) individuals. For the remaining 30 individuals without adult EEG data, the DEE diagnosis was made on evaluation of phenotype at the time of previous (non-adult) EEG studies. For the individuals with adult EEG data, the prevalence of EE was 69.7% (69/99 assessable individuals), with 38 genes implicated. EEG and clinical features in adulthood characteristic of Lennox–Gastaut syndrome^40^ were present in only one individual (with *LIS1*-related DEE). Cohort-specific prevalence was 63.4% (26/41 individuals) for the *SCN1A* and 86% (43/50 individuals) for the non-*SCN1A* cohort.

The remaining 30/99 (30.3%) individuals (non-EE group) either had (i) seizures and encephalopathy (slow EEG background) without epileptiform activity or with epileptiform activity but without clinical seizures (16/99 individuals; 16.1%); (ii) no encephalopathy (reactive background rhythms) with ongoing interictal epileptiform activity ± seizures (8/99 individuals; 8.1%); or (iii) had a normal EEG with or without clinical seizures (6/99; 6.1%). Cohort specific prevalence for each clinical and EEG phenotype is detailed in Table 1.

**Table 1:** Clinical and EEG phenotypes across the DEE cohort

Clinical and EEG phenotypes	*SCN1A* cohort; point prevalence *N* (%)	Non-*SCN1A* cohort; point prevalence *N* (%)	Genes (for non-*SCN1A* cohort)
Epileptic encephalopathy	26/41 (63.4)	43/58 (74.1)	***ANKRD11*** *(2)*, *AP4M1*, *AP4S1*, *ATP1A2*, ***CHD2****(4)*, *COL4A1 (2)*, *DEAF1*, *DNM1*, ***KCNA2****(2)*, ***KCNQ2***, *KCNT1 (3)*, *NEXMIF (3)*, *LIS1*^a^, *MECP2 (2)*, *PCDH19*, *PTEN*, *PURA (4)*, *SATB1*, *SCN2A*, *SCN8A*, *STED1B*, *SETD5 (2)*, *SLC6A1*, *SMS*, *STXBP1*, *UBE3A (2)*
Encephalopathy	9/41 (22)	7/58 (12.1)	*CAMK2A*, *COL4A2*, *DNMT3A*, *GAMT*, *GNAO1*, ***KCNA2***, ***KCNQ2***
No encephalopathy	3/41 (7.3)	5/58 (8.6)	***ANKRD11***, ***CHD2***, *KDM3B*, *GABRA1*, *KIF1A*
Within normal limits	3/41 (7.3)	3/58 (5.2)	*CNTNAP2*, *SOX2* (2)

EEGs in adulthood were available for 99/129 (76.6%) individuals: 41/99 from the *SCN1A* cohort and 58/99 from the non-*SCN1A* cohort. Genes in bold are those appearing in more than one category. The parentheses in the fourth column indicate the number of individuals involved when greater than one. ^a^The individual with *LIS1*-related DEE had EEG and clinical features of Lennox–Gastaut syndrome.^38^

**Figure 4:**
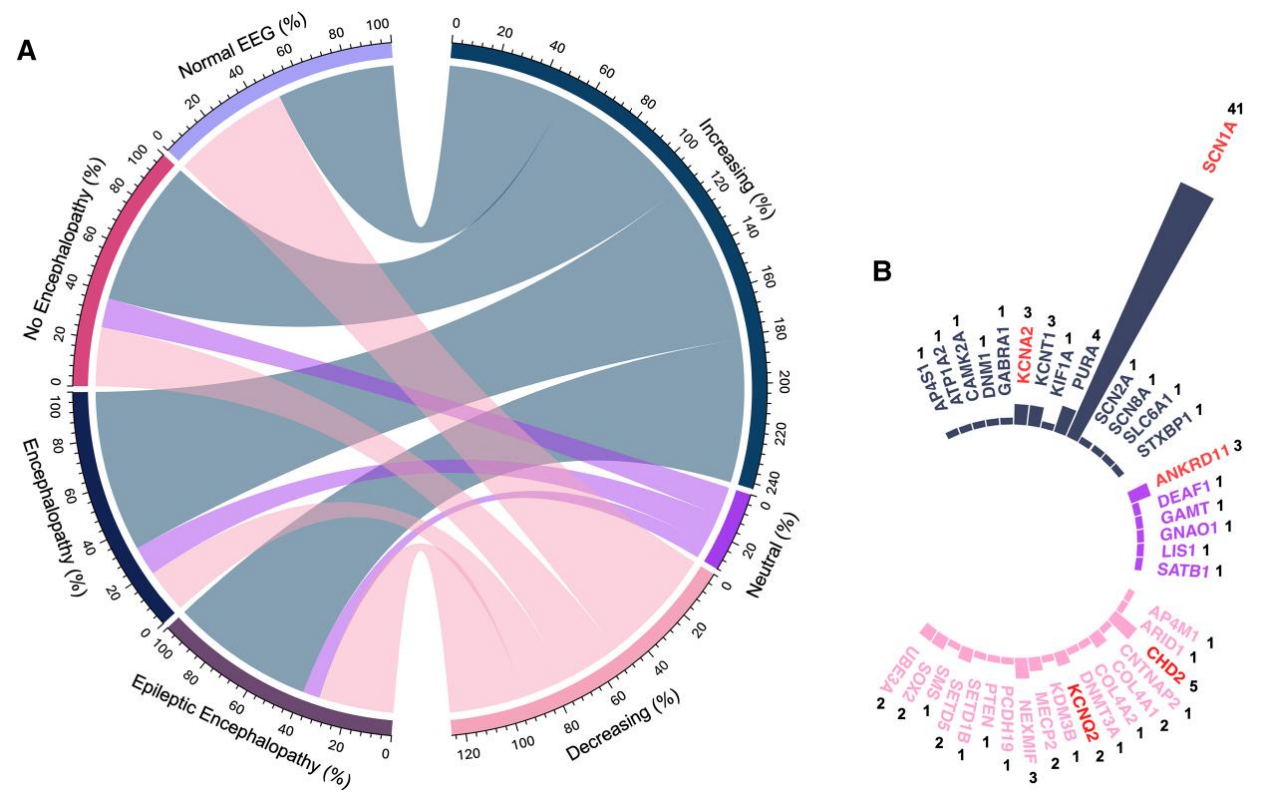
**Temporal gene expression trends and their association with clinical and EEG phenotypes across the cohort.** (**A**) Chord diagram illustrating the prevalence of different gene expression trends, based on the number of individuals involved (depicted in the semi-circle on the right-hand side of **A**), and their association with the reported clinical and EEG phenotypes across the entire cohort (depicted in the semi-circle on the left-hand side of **A**). No statistically significant association was found between the temporal trends of gene expression and the clinical and EEG phenotypes [Fisher’s exact test (*n* = 99), *P* = 0.75]. (**B**) Circular bar plot detailing the specific genes involved across the different expression trends from **A**. The colours of the bars correspond to the colours of the gene expression trends shown in **A** (dark blue: increasing gene expression, purple: neutral gene expression, pink: decreasing gene expression). Each bar represents the number of individuals involved for each specific gene, as indicated by the number at the end of each bar. Genes labelled in red font are those observed across different clinical and EEG phenotypes; details of the clinical and EEG phenotypes and the specific genes involved can be found in Supplementary Table 3.

The prevalence of each gene expression trend across all described clinical and EEG phenotypes is shown in Fig. 4, with further details of the specific genes implicated in each clinical and EEG phenotype in Supplementary Table 3.

**Figure 5:**
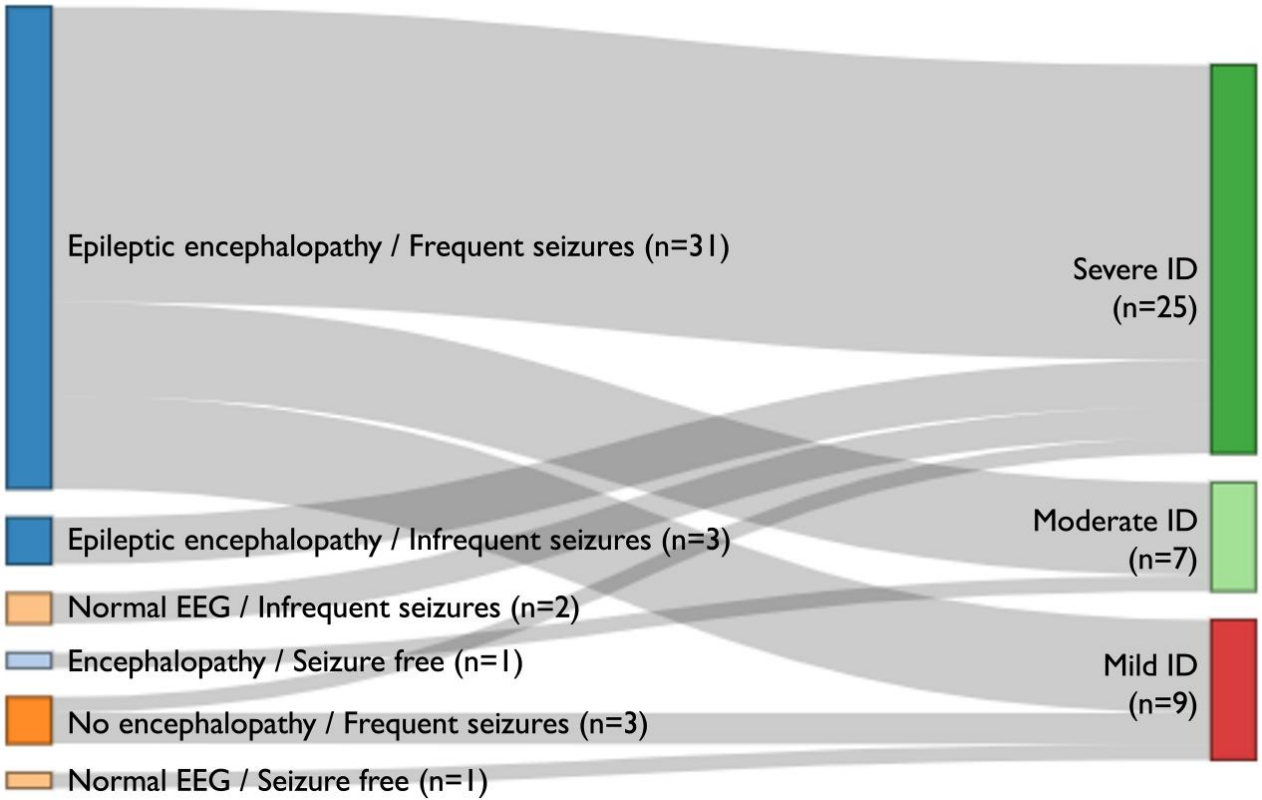
**Clinical and EEG and cognitive phenotypes across the *SCN1A* cohort at last follow-up.** This diagram illustrates the spectrum of clinical and EEG phenotypes observed in individuals with *SCN1A*-related epilepsies and their relationship with their reported cognitive phenotypes. Complete EEG, seizure frequency and cognitive data were available for 41/59 (69.5%) individuals, all of whom had a diagnosis of Dravet syndrome. Various clinical and EEG phenotypes were observed ranging from EE with or without frequent seizures to seizure-free individuals with normal EEG studies. While severe ID was prevalent among individuals with EE [19/31 (61.3%) of individuals with EE and frequent seizures and 3/3 (100%) of individuals with EE and infrequent seizures], significant variability was evident with individuals exhibiting severe ID across less severe clinical and EEG phenotypes, such as those with normal EEG and infrequent seizures. ID, intellectual disability; EE, epileptic encephalopathy.

A diverse range of phenotypes, concerning EEG features, seizures and intellectual abilities, was observed in the *SCN1A* cohort (Fig. 5). Similarly, significant phenotypic heterogeneity was observed among the non-*SCN1A* cohort, albeit the sample size is small for each gene-specific DEE (Supplementary Figs. 3–5).

### ASMs and management changes post-genetic diagnosis

Information on treatment and other clinical management changes was available for 124/129 (96.1%) individuals. Therapeutic interventions guided by the genetic diagnosis, and based on the pharmacological options available at the time of diagnosis, were implemented for 68/124 (54.8%) individuals, including ASM changes (*n* = 28; 22.6%) and/or specialist referral for screening of clinical manifestations related to the specific genetic DEE (*n* = 47; 37.9%; Supplementary Table 4).

#### *SCN1A* cohort

Information on management changes was available for 54/59 (91.5%) individuals, of which 40 (74.1%) underwent changes, including ASM change (*n* = 18), further specialist referral (*n* = 15) or both (*n* = 7). The median number of ASMs tried was eight (IQR: 7–11); the median number of ASMs at last follow-up was three (IQR: 2–3).

#### Non-*SCN1A* cohort

Information on management changes was available for all individuals, with 3/70 (4.3%) receiving a genetically-informed ASM change (*KCNA2*, *SCN2A* and *SLC2A1*-related DEEs) and with 25/70 (35.7%) receiving a post-diagnosis specialist referral. The median number of ASMs tried was seven (IQR: 5–10); the median number of ASMs at last follow-up was three (IQR: 2–3).

### Cognitive and behavioural phenotypes

#### *SCN1A* cohort

Formal neuropsychometry was conducted in 15/59 (25.4%) individuals. In the remaining, level of intellectual function was documented in the medical records by the treating clinician. All 59 individuals had ID at last follow-up, with various degrees of language impairment (Fig. 2). Twenty out of 59 individuals (33.9%) were either diagnosed with autism spectrum disorder (ASD; *n* = 15) or exhibited autistic features (*n* = 5). Two individuals (3.4%) were diagnosed with attention deficit hyperactivity disorder (ADHD). Behavioural and psychiatric problems were reported in 27/59 (45.8%) individuals, including aggressive behaviour (*n* = 22; 37.3%), depression/anxiety (*n* = 4; 6.8%), and psychosis (*n* = 1; 1.7%).

#### Non-*SCN1A* cohort

Formal neuropsychometry was conducted in 24/70 (34.3%) individuals. In the remaining, level of intellectual function was documented in the medical records by the treating clinician. Only one individual (1/70, 1.42%), with a diagnosis of *ANKRD11*-related KGB syndrome, had borderline intellectual abilities, with the remaining having more severe impairments (Fig. 3). The prevalence of ASD or ADHD along with types and prevalence of psychiatric comorbidities are detailed in Table 2.

**Table 2:** Behavioural phenotypes and psychiatric comorbidities across the non-*SCN1A* (*n* = 70) cohort

Behavioural phenotypes and psychiatric comorbidities	Point prevalence *N* (%)	Genes
ASD^a^	24/70 (34.3)	***ANKRD11*** (3), ***CAMK2A***, ***CHD2*** (3), *CNTNAP2*, ***COL4A1***, *DEAF1*, ***KCNQ2*** (2), ***KCNT1*** (2), *KDM3B*, *NEXMIF* (2), ***KIF1A***, ***PCDH19***, *PTEN*, *SCN2A*, *SETD1B*, *SETD5*, *SMS*
ADHD	3/70 (1.4)	***CAMK2A***, ***KDM6A***, ***KCNT1***^b^
Aggressive behaviour	9/70 (12.9)	***ANKRD11*** *(2)*, ***COL4A1****(2)*, *COL4A2*, *GAMT (2)*, ***PCDH19***, *PTEN*
Anxiety/depression	4/70 (5.7)	***ANKRD11***, ***CAMK2A***, ***KCNT1***, ***KDM6A***
Psychosis	2/70 (4.3)	***CHD2***, *SLC2A1*
OCD	2/70 (4.3)	*DNMT3A,* ***KIF1A*** ^b^
Post-ictal psychosis	1/70 (1.4)	*SOX2*
Bipolar disorder	1/70 (1.4)	***KCNQ2***
Non-epileptic seizures	1/70 (1.4)	*SLC6A1*

Genes in bold are those appearing in more than one category. The parentheses in the fourth column indicate the number of individuals involved when greater than one. ADHD, attention deficit hyperactivity disorder; ASD, autism spectrum disorder; OCD, obsessive compulsive disorder. ^a^19/24 (79.2%) individuals had a formal diagnosis of ASD, whereas the remaining five exhibited autistic features but had no formal diagnosis of ASD. ^b^Individuals with a reported psychiatric comorbidity but without a formal psychiatric assessment.

### Mobility, feeding, ADLs

#### *SCN1A* cohort

Whilst the majority remained independently ambulant at last follow-up (52/59; 88.1%), mobility deficits were described in 37/59 (62.7%), including crouch gait (30/37; 81.1%) and ataxia (7/37; 18.9%). Feeding difficulties were reported in 35/59 (59.3%) individuals comprising anorexia and/or weight loss (26/35; 74.3%), dysphagia (13/35; 37.1%) or other feeding difficulties (2/35; 5.7%), with 7/35 (20%) individuals exhibiting more than one feeding difficulty. Of the 56/59 (94.9%) individuals that were fully dependent for ADLs, 17/56 (30.3%) were living in residential care settings and the remaining (39/56, 69.7%) were living at home with family and/or additional support from professional carers (Fig. 2).

#### Non-*SCN1A* cohort

Of the 17/29 (58.6%) individuals who were wheelchair-bound at last follow-up, six (35.3%) had been wheelchair-bound since early childhood [AP4M1, AP4S1, DNM1, LIS1 and PURA (*n* = 2)-related DEEs]. Dysphagia was the most prevalent difficulty in the individuals with reported feeding difficulties (10/12, 83.3%). Of the 59/70 (84.3%) individuals who were fully dependent for ADLs, 17/59 (28.8%) were living in residential care settings, with the remaining 42/59 (71.2%) living at home with family with or without additional support from professional carers (Fig. 3).

### Neurological and non-neurological comorbidities

Various neurological and non-neurological comorbid conditions were documented across both *SCN1A* and non-*SCN1A* cohorts. The prevalence estimates described are likely to be underestimates as systematic questioning and thorough evaluation had often not been conducted.

#### *SCN1A* cohort

The most prevalent neurological comorbidities were ataxia and crouch gait, documented in 7/59 (11.9%) and 30/59 (50.8%), respectively; followed by spasticity in 3/59 (5.1%), extra-pyramidal signs (tremor, dystonia, rigidity) in 3/50 (5.1%), sialorrhea in 2/59 (3.4%), migraine/headache in 2/59 (3.4%) and ptosis in 1/59 (1.7%).

Non-neurological comorbidities were reported in 26/59 (44%) individuals, with 9/26 (34.6%) individuals having more than one comorbidity. Musculoskeletal comorbidities were described in 13/59 (22%), with scoliosis being most prevalent (10/13, 77%) followed by osteoporosis (3/13, 23%). Cardiological comorbidities were the second commonly reported (4/59, 6.8%), followed by gastrointestinal comorbidities (3/59, 5%; Supplementary Fig. 1).

#### Non-*SCN1A* cohort

Ataxia was the most commonly documented neurological comorbidity (6/70; 8.6% in *ANKRD11*, *CNTNAP2*, *KCNA2*, *KCNQ2*, *SCN2A* and *STXBP1*-related DEEs), followed by microcephaly [6/70; 8.6% in *AP4M1* (*n* = 2), *AP4S1*, *CHD2*, *IQSEC2*, *SCN2A*-related DEEs], dyspraxia (5/70; 7.1% in *ANKRD11*, *GAMT*, *KCNT1*, *KDM3B*, *SLC2A1*, *TBC1D24*-related DEEs), quadriparesis/hemiparesis (4/70; 5.7% in *COL4A1*, *KDM6A*, *SCN8A*, *TBC1D24*-related DEEs), extra-pyramidal signs (4/70; 5.7% in *KCNQ2*, *GNAO1*, *SCN2A*, *SLC2A1*-related DEEs), sialorrhea (3/70; 4.3% in *AP4M1*, *IQSEC2*, *KCNA2*-related DEEs), spasticity (2/70; 2.9% in *MECP2*, *SATB1*-related DEEs), dysarthria (2/70; 2.9% in *ARID2*, *KCNA2*-related DEEs) and single cases (1/70; 1.4%) of migraine (*SLC6A1*-related DEE), cortical blindness (*PURA*-related DEE), congenital hydrocephalus (*KIF1A*-related DEE), hyperkinetic movement disorder (*MECP2*-related DEE), hypotonia (*STXBP1*-related DEE), limb hemi-atrophy (*SLC2A1*-related DEE), dysautonomia (*GNAO1*-related DEE), neuropathic bladder (*SCN2A*-related DEE), and astrocytoma (*KDM6A*-related DEE).

Non-neurological comorbidities were reported in the majority (42/70, 60%), with 21/42 (50%) individuals having more than one comorbidity. Musculoskeletal comorbidities were the most commonly reported (18/70, 25.7%), with scoliosis being most prevalent (14/18, 77.8%), followed by osteoporosis (5/18, 27.8%) and hip dislocation (3/18, 16.7%). Gastrointestinal comorbidities were reported in 16/70 (22.9%) with gastro-oesophageal regurgitation and constipation being most prevalent, described in 7/16 (43.7%) and 4/16 (25%), respectively (Supplementary Fig. 1).

### Mortality

Eight individuals (6.2%) had died after their last follow-up; four had *SCN1A*-related Dravet syndrome; the others had *CHD2*, *GNAO1*, *KCNT1* and *NEXMIF*-related DEEs. The mean age of death was 45.9 years (SD: 14.1).

### Associations of outcomes

There was no significant difference in age of seizure onset between the different cognitive and functional phenotypes of interest. The only nominally significant association between seizure phenotypes and cognitive or functional phenotypes was between seizure frequency and level of dependence for ADLs: χ^2^ (1, *n* = 129) = 4.93, *P* = 0.02, but the significance was lost following Bonferroni correction. A statistically significant association was found between clinical and EEG phenotypes (presence of EE) and cognitive phenotypes, χ^2^ (1, *n* = 99) = 11.4, corrected *P* = 0.0007; OR = 0.17, 95% CI 0.06–0.47, indicating that the presence of EE is associated with poorer cognitive phenotypes.

No significant association was found between the temporal trends of gene expression and the presence of EE.

### Disease progression

In the absence of systematic longitudinal data, aspects of disease progression, where available, are presented based on the reports from treating clinicians, families and carers as documented in early medical records.

In the *SCN1A* cohort, seizure frequency improved in 33/59 (55.9%) individuals, notably following genetic diagnosis and treatment adjustments. Gradual cognitive decline was observed in 8/59 (13.5%) individuals. Cognitive improvement was reported in 6/59 (10.2%), reportedly corresponding to better seizure control in five individuals; in one individual, improvement was noted at different time points following either improved seizure control or change in residential care setting. While progressive deterioration of behavioural problems was reported in 5/42 (11.9%) individuals, no change was reported in most cases. Mobility declined progressively in 33/37 (89.2%) individuals and feeding difficulties worsened in all 20 cases with reported issues (Supplementary Table 5).

In the non-*SCN1A* cohort, seizure deterioration was reported in 21/70 (30%) individuals, with variable responses to treatment across different genetic subtypes. Notably, seizure deterioration was reported in 100% of individuals with *NEXMIF* (3/3), *MECP2* (3/3)-related DEEs and in 50% of individuals with *PURA* (2/4) and *CHD2* (3/6)-related DEEs. Cognitive decline was noted in 5/70 (7.1%) individuals, with sporadic improvement during periods of improved seizure control observed in some cases (*CHD2*, *MECP2* and *KCNQ2*-related DEEs). Only one individual showed cognitive improvement, and this was associated with improved seizure control (*CAMK2A*-related DEE). Behavioural problems progressed in 4/33 (12.1%) individuals with psychiatric comorbidities, while stability prevailed in the remainder. Mobility deterioration was reported in 16/29 (55.2%) individuals with difficulties. Swallowing function worsened in 9/12 (75%) individuals who had any reported difficulties (Supplementary Table 5).

Details of ASM treatment over time for both cohorts can be found in Supplementary Material 1, Supplementary Fig. 2.

Of the 99 individuals with adult EEGs studies, 24 (24.2%) had accessible childhood EEG data. EEG features between childhood and adulthood are detailed in Supplementary Material 2 and Supplementary Table 6. Eight individuals had only childhood EEG data, details of which can be found in Supplementary Material 3.

## Discussion

We examined the adult phenotypes of genetic DEEs. Irrespective of the culpable gene, all individuals studied typically had several difficulties comprising drug-resistant epilepsy, ID, multi-morbidities, impairments in mobility, feeding and verbal communication, and a high level of dependency for ADLs. The majority of the assessable described DEEs (69.7%) are EEs in adulthood, with persistent seizure burdens contributing to the clinical state according to our current conceptualization of the epileptic component of these conditions. However, our data emphasize the complexity in factors contributing to the clinical outcomes of DEEs in adulthood. Whilst the presence of EE was significantly associated with poorer cognitive outcomes, the relationship between cognitive and clinical and EEG phenotypes was nuanced, even for seizure-free individuals, or even when individual genes were examined, and across the spectrum of age-related trends in gene expression. These findings highlight the intricate interplay of factors influencing the adult DEE phenotype. Focusing primarily on seizures and EEG data when outcomes are concerned risks oversimplifying the complexity of these conditions, which may lead to inadequate healthcare provision.

Drug-resistant epilepsy with frequent seizures was common in both *SCN1A* and non-*SCN1A* cohorts, with most individuals experiencing at least monthly seizures, whilst only 6.2% across the entire cohort were seizure-free at last follow-up. Consequently, ASM polytherapy, with three or more ASMs at last follow up, was common in both cohorts.

ID, a hallmark of genetic DEEs,^41^ is a consistent feature in adulthood encompassing all DEEs described here. While no significant association was detected between the ongoing occurrence of seizures alone and cognitive phenotypes, those with an ongoing EE were more likely to have worse cognitive phenotypes (with moderate/severe/profound, as opposed to mild ID).

Despite this association, the relationship between the cognitive and clinical and EEG phenotypes (including non-EE) proved complex. For example, among seizure-free individuals (*n* = 6), cognitive outcomes varied: two had normal EEG and mild ID (*SCN1A* and *SOX2*-related DEEs), three had encephalopathy (background slowing) without epileptiform activity and moderate or severe ID (*SCN1A*, *DNMT3A* and *KCNQ2*-related DEEs), and one had encephalopathy with ongoing interictal epileptiform activity and moderate ID (*CAMK2A*-related DEE). Another individual, with *KCNA2*-DEE (gain-of-function phenotype), who had encephalopathy (background slowing, no epileptiform activity, rare clinical seizures) and mild ID, exhibited a progressive decline in cognition and mobility. Furthermore, for seven individuals, cognitive improvement was observed with better seizure control, but this did not correlate with the overall outcome: at last follow up, five still had EE (with ID spanning from mild to severe), one had encephalopathy and moderate ID (*CAMK2A*-related DEE) and one had normal EEG and severe ID. These findings suggest that while optimizing seizure control is crucial, it may not suffice to improve overall outcomes, underscoring the complexities of DEE outcomes in adulthood. Recent data have similarly demonstrated a broad range of outcome severity in paediatric populations too, with respect to cognitive and EEG outcomes.^42^

Studies investigating the association between seizures and cognitive outcomes in adults are sparse and often focus on cognitive outcomes in relation to seizures alone rather than the combined impact of seizures and EEG features. In adults with *STXBP1*-related DEEs, no correlation between the seizure and cognitive outcomes was reported.^12^ In adults with *KCNQ2*-DEEs, ID persisted despite seizure remission.^15^ For adults with *SCN1A*-related Dravet syndrome, worse intellectual outcomes correlated with persisting seizures, yet improved seizure control did not necessarily lead to cognitive improvement (accepting that scales used to measure improvement may not be sufficiently sensitive), corroborating our findings, that seizures may not be the sole determinant of outcomes.^43^ The factors influencing this diversity in adult phenotypes remain to be elucidated and may include at least the strength of impact of the driving genetic variant, background genomic variation,^44^ early-life seizure frequency, ASM polypharmacy and appropriateness of used ASMs, and non-ASM treatments with impact on cognition such as antipsychotic medications. Additionally, the care setting, either residential or home-based—or more likely, the nature of the living environment, appropriateness and amount of stimulation provided^45^—and normal aging, may also play a role in shaping cognitive outcomes. A holistic treatment approach for DEEs should combine rigorous seizure management and aetiology-specific gene-targeted treatment, where available. In the absence of readily-available gene-targeted therapies for most DEEs currently, optimizing seizure management remains crucial, due to the significant health risks of uncontrolled seizures, including physical injuries, the potential for sudden unexpected death in epilepsy,^46^ and a deleterious impact on overall quality of life, even as we also take into account the need to minimize potential adverse (including cognitive) side effects.

We did not identify any association between age-related trends (and thus presumed persistence or otherwise) of gene expression and clinical and EEG or cognitive phenotypes, which may relate to the small cohort size for most of the involved genes. Clinical and EEG features indicating an encephalopathy were seen across various genes and temporal gene expression trends (Fig. 4). While non-genetic epilepsy models show seizures can alter gene expression involved in neuronal signalling and plasticity, evidence from genetic epilepsy models is limited.^47-50^ One study on an *SCN8A* encephalopathy mouse model demonstrated altered gene expression after seizures but not in the causative gene itself.^51^

Additionally, there was a diverse spectrum of observed clinical and EEG and cognitive phenotypes for individuals with pathogenic variants in a single gene, as illustrated by the *SCN1A* cohort (Fig. 5). A degree of variability was also observed for other genetic DEEs for which we had at least more than one individual carrying a variant in that gene (*KCNA2*, *KCNT1*, *NEXMIF*, *CHD2*, *MECP2*, *STED5*, *SOX2*, *ANKRD11*-related DEEs), though for some genes, more homogenous phenotypes were seen (*PURA*, *UBE3A*, *COL4A2*-related DEEs) (Supplementary Figs. 3–5). Our findings mainly demonstrate phenotypic variability even for individual gene-driven DEEs, though some DEEs may exhibit a higher degree of homogeneity. The small sample size for each DEE limits our ability to elucidate patterns or draw robust conclusions with respect to the timing of change in their expression trajectories, which might have offered guidance on the optimal timing of gene-targeted therapies, as such therapies may be ineffective past certain points in the disease trajectories or gene expression profiles. While genomics has significantly improved the diagnostics of DEEs, the biology of their temporal evolution remains complex, even for DEEs due to variants in single genes or variant-effect types (e.g. loss-of-function). For some genetic DEEs, the variation in outcomes may involve factors beyond features examined here, such as the variation across the rest of the genome, as has been shown for *SCN1A*-related Dravet syndrome and other rare genetic neurological conditions.^44,52,53^ These nuances highlight the potential complexity in developing specific gene-based disease-modifying treatments. Our data show that at least some DEEs remain dynamic conditions in adulthood, with potential for improved outcomes, stressing the need for the inclusion of adults in treatment trials, including those of precision medicines, a practice often currently lacking.^54-56^

Striking delays were observed in making a genetic diagnosis, with an average of 25.2 years between seizure onset and genetic diagnosis, confirming previous reports that people who are adults today, especially those with drug-resistant epilepsy and ID, have usually (and often inevitably) missed out on the benefits of early utilization of advanced sequencing technologies.^11^ The clinical utility of genetic diagnoses is increasingly recognized, with recent data showing that genetically-informed treatments are feasible in 56% of paediatric cases and additional specialist evaluations in 65%.^57^ Importantly, even in adulthood, a genetic diagnosis can lead to changes in clinical management in up to 50% of adult individuals.^11^ For our cohort, despite the long diagnostic delay, disease-specific treatment adjustments were made in 54.8%, either in the form of genetically-informed ASM adjustment (primarily described in the S*CN1A* cohort) or, equally important for these complex conditions, specialist referral for screening of non-neurological clinical manifestations related to the specific genetic syndrome. This indicates the importance of considering genome-wide genetic testing (e.g. exome or genome sequencing) in adults with compatible phenotypes, as a genetic diagnosis cannot be excluded on the basis of history alone, and in many cases, the early history may be unavailable, or the history overall may not suggest a particular genetic diagnosis.^4,58^ All the individuals in this study would now be eligible—in adulthood—for whole genome sequencing through the UK’s National Health Service (NHS).^59^

The DEEs described here are often multisystem conditions, with broad clinical manifestations in adulthood, including mobility, feeding and verbal communication deficits and prevalent behavioural and psychiatric difficulties. The prevalences of neurological and non-neurological comorbidities reported here are likely underestimates, due to the lack of systematic documentation in the medical records. Neurological abnormalities beyond seizures were common, though the relationship between these neurological features and specific genetic DEEs remains unclear given the range of implicated genes and the limited number of individuals per gene. Non-neurological comorbidities were also described; musculoskeletal problems were the most prevalent in both cohorts, followed by cardiological conditions in the *SCN1A* and gastrointestinal problems in the non-*SCN1A* cohort. Additionally, the vast majority was dependent on others for their basic ADLs, with a significant proportion requiring continuous care either in residential or home settings. These findings underscore the necessity for lifelong high levels of support, for issues beyond seizures alone: insufficient provision itself compromises quality of life.^60^ Many DEEs are often labelled ‘childhood epilepsies’, sometimes leading to challenges in transitioning into adult care services.^61^ Effective transition services are important for reducing long-term morbidity and mortality.^62,63^ Factors such as ID and limited knowledge of the natural history of complex conditions^64,65^ can affect the quality of these transitions and adult care.

Our study has important limitations. This study is cross-sectional: disease progression was not its primary focus. Ongoing prospective studies are addressing this aspect,^66,67^ albeit inevitably at the pace of real time. Cross-sectional studies still offer valuable insights into adult phenotypes of these epilepsies. Although contemporaneous records were used, clinical data collection typically did not employ standardized scales. In many cases, clinical data, particularly for early history, were sparse, especially regarding cognitive or language assessments and early seizure types classified using current systems. Diagnoses regarding cognitive phenotypes, psychiatric and other comorbidities were collected based on the reports of treating clinicians as specific information was typically unavailable. We also acknowledge that many aspects may have been under-ascertained, as specific questioning, examination or evaluation had often not been undertaken. These observations argue for specialist service provision with systematic management approaches. Regarding the temporal trends of gene expression, we assume that the trend for a single gene is homogenous across individuals.^68^ Furthermore, the majority of gene expression studies have been conducted in normal brain tissues, with limited data available from brain tissues of individuals harbouring pathogenic variants. Additionally, it is important to acknowledge that we have not elucidated the specific aspects of gene expression that exert an influence on the phenotype; rather, we have employed a rudimentary measure of gene expression itself. Our cohort was highly skewed towards *SCN1A*-related DEEs, due both to our local interest in these genetic syndromes and to their relatively high frequency.^69^ The individuals presented were all seen in a highly specialized epilepsy tertiary centre, which will have introduced some ascertainment bias towards the more severe DEE phenotypes.

## Conclusion

The DEEs described here appear to be EEs in adulthood, with only a minority exhibiting seizure freedom or normal EEGs and with significant heterogeneity in clinical features, even within gene-specific DEEs. The significant link between the presence of EE and poorer cognitive outcomes highlights the ongoing importance of optimizing seizure control throughout adulthood. The variability in outcomes may vary even with optimal seizure control, underscoring the need for treatments beyond seizure management and the importance of expanding the use of newer therapies for individuals, across the DEE spectrum, ensuring that adults and children are equally considered. Understanding the complexity of diverse molecular pathways and their spatiotemporal patterns is crucial for developing and administering such therapies in a timely manner.

## Supplementary Material

fcaf028_Supplementary_Data

## Data Availability

Anonymized statistical data related to the main findings are available from the corresponding author upon reasonable request from any qualified investigator subject to the necessary agreements. Other data are not available as they contain information that could compromise the privacy of research participants. No bespoke code was used for this study. All code used in the manuscript is in the public domain already and has been appropriately referenced.
