# Supplementary material for: Adult phenotypes of genetic developmental and epileptic encephalopathies
Source: Brain Commun. 2025 Jan 20;7(1):fcaf028. doi: 10.1093/braincomms/fcaf028 (PMC11775618; doi:10.1093/braincomms/fcaf028)
Supplement: fcaf028_Supplementary_Data [file fcaf028_supplementary_data.pdf]

Supplementary Information:

1) **Supplementary Materials**
2) **Supplementary Figures**
3) **Supplementary Tables**

Abbreviations: ACZ: acetazolamide; ASM: antiseizure medication; BRO: bromide, BRV: brivaracetam, CBD: cannabidiol; CBZ: carbamazepine; CLB: clobazam; CLZ: clonazepam; DZP: diazepam; ESZ: eslicarbazepine; ETX: ethosuximide; FBM: felbamate; GBP: gabapentin; IEDs: interictal epileptiform discharges; KD: ketogenic diet; LCS: lacosamide; LEV: levetiracetam; LTG: lamotrigine; LZP: lorazepam, NTZ: nitrazepam; OXC: oxcarbazepine; PB: phenobarbital; PCT: piracetam; PDX: pyridoxine; PER: perampanel; PGB: pregabalin; PHT: phenytoin; PIR: piracetam; PMD: primidone; RFD: rufinamide; RTG: retigabine; STP: stiripentol; SUL: sulthiame; TPM: topiramate; TGB: tiagabine; VGB: vigabatrin; VPA: valproate; ZNS: zonisamide

1. Supplementary Materials

Supplementary Material 1: Antiseizure medications (ASMs) across the cohort

For the *SCN1A* cohort (n=59), the most commonly prescribed ASM at last follow-up was valproate (VPA), followed by clobazam (CLB) and levetiracetam (LEV) (Supplementary Fig. 2A). Notably, ASM exposure over the entire epilepsy trajectory showed that sodium channel-blocking medications had been prescribed in the majority of these individuals, with lamotrigine (LTG) and carbamazepine (CBZ) trialled in 37/59 (62.7%) and 35/59 (59.3%) respectively (Supplementary Fig. 2B). Information on ASMs that clearly improved and/or worsened seizure control throughout the epilepsy course (if any) was available for 42 individuals; VPA was reported to have improved seizure control most frequently (11/41 exposed to VPA; 26.8%), followed by STP (6/17 exposed to STP; 35.3%) and ketogenic diet (KD) (4/11 exposed to KD; 36.3%). CBZ and LTG were most frequently reported culprit ASMs for seizure aggravation, described in 14/30 (46.7%) and 10/30 (33.3%) individuals respectively. Since the non-*SCN1A* cohort

predominantly consists of singe-individual cases, treatment information over time has been excluded to minimise the risk of identification.

Supplementary Material 2: EEG studies from childhood and adulthood

Of the 24/99 (24.2%) individuals with EEG studies from childhood and adulthood, 12/24 (50%) had *SCN1A*-related DEEs. For these 12 individuals, EEG between childhood and adulthood showed overall unchanged features with evidence of encephalopathy and epileptiform activity in eight individuals. Features of photosensitivity in childhood but not in adulthood were reported in one out of the eight individuals. For one individual, reactive background rhythms with epileptiform activity were seen consistently both in childhood and adulthood EEG. For two individuals, EEG in childhood was within normal limits but showed evidence of encephalopathy with epileptiform activity in adulthood, whereas one individual EEG was within normal limits in both childhood and adulthood (Supplementary Table 6).

For the remaining 12 individuals with other genetic DEEs, EEG studies were similar between childhood and adulthood, with epileptic encephalopathy in four or encephalopathy without epileptiform activity in three individuals. Improvement of EEG was seen in one individual, with a normal EEG study in adulthood, having been encephalopathic in childhood. Worsening of EEG was seen in two individuals, with normal EEG in childhood but with evidence of epileptic encephalopathy or frequent epileptiform activity without encephalopathy in adulthood (Supplementary Table 6). Hypsarrhythmia and EEG features compatible with Lennox-Gastaut syndrome were reported in childhood EEGs for two individuals, with evidence of epileptic encephalopathy but without features of Lennox-Gastaut syndrome in adulthood (Supplementary Table 6). To minimise risk of identification, details on the specific genes involved have not been provided.

Supplementary Material 3: EEG studies exclusively from childhood

Of the eight individuals with EEG studies available from childhood only, five had a diagnosis of *SCN1A*-related Dravet syndrome: one had an epileptic encephalopathy, whereas in two there was evidence of encephalopathy without epileptiform activity. For the remaining two, one had epileptiform activity on EEG, without information on EEG

rhythms and in one the EEG was within normal limits. The remaining three individuals had encephalopathic EEG with epileptiform abnormalities.

2. Supplementary Figures

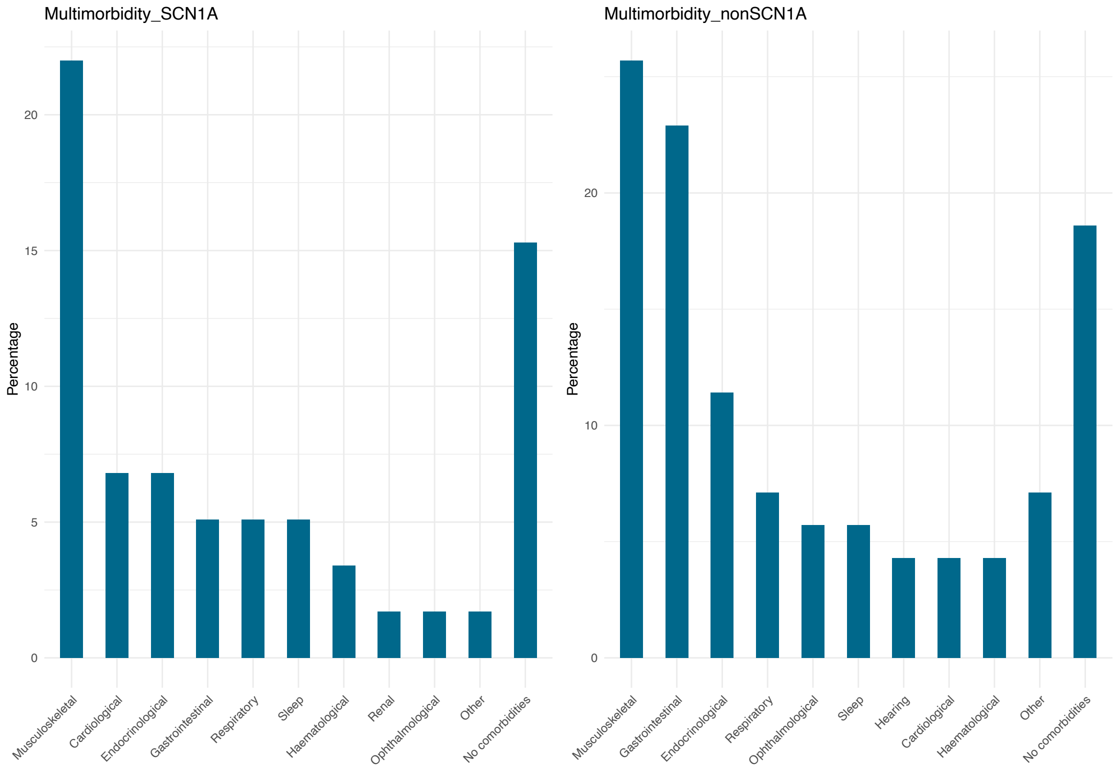

Supplementary Fig. 1. Types and prevalence of comorbidities across both *SCN1A* (n=59) and non-*SCN1A* (n=70) cohorts. Information about multimorbidity was taken from the information as documented by treating clinicians in the medical records.

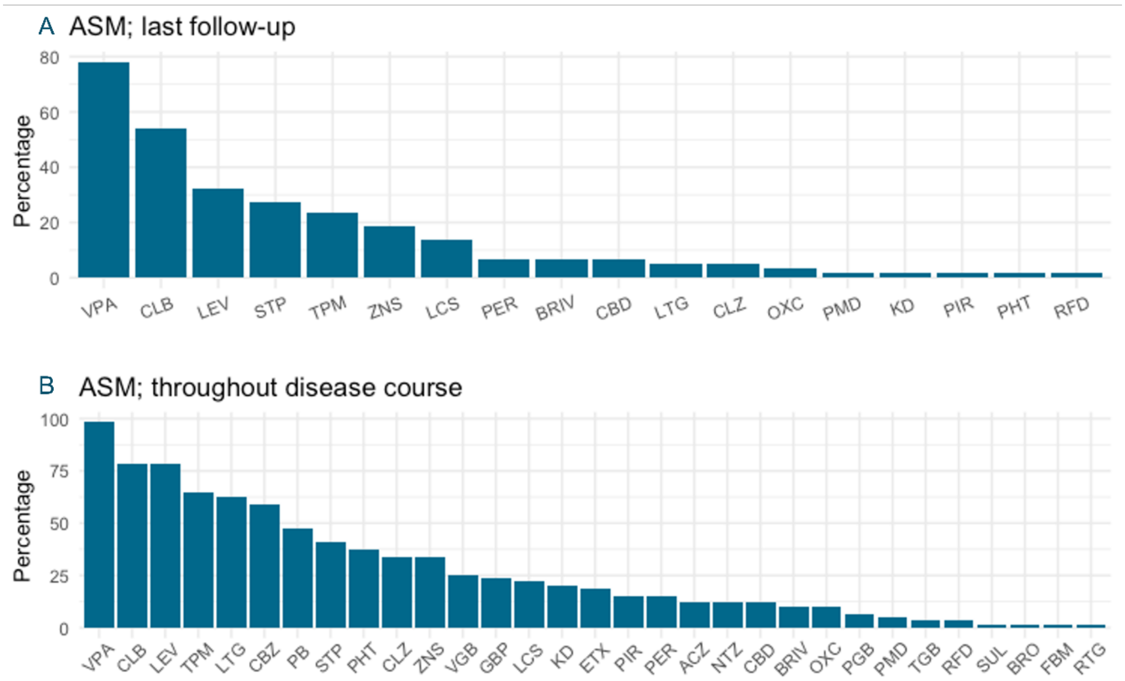

Supplementary Fig. 2. Antiseizure treatment (ASM) for the *SCN1A* cohort (n=59).

A. ASMs at last follow-up. **B.** ASM history throughout disease course.

Abbreviations: VPA: valproate; CLB: clobazam; LEV: levetiracetam; STP: stiripentol; TPM: topiramate; ZNS: zonisamide; LCS: lacosamide; PER: perampanel; BRV: brivaracetam; CBD: cannabidiol; LTG: lamotrigine; CLZ: clonazepam; OXC: oxcarbazepine; PMD: primidone; KD: ketogenic diet; PIR: piracetam; PHT: phenytoin; RFD: rufinamide; CBZ: carbamazepine; PB: phenobarbital; VGB: vigabatrin; GBP: gabapentin; ETX: ethosuximide; ACZ: acetazolamide; NTZ: nitrazepam; PGB: pregabalin; TGB: tiagabine; SUL: sulthiame; BRO: bromide; FBM: felbamate; RTG: retigabine

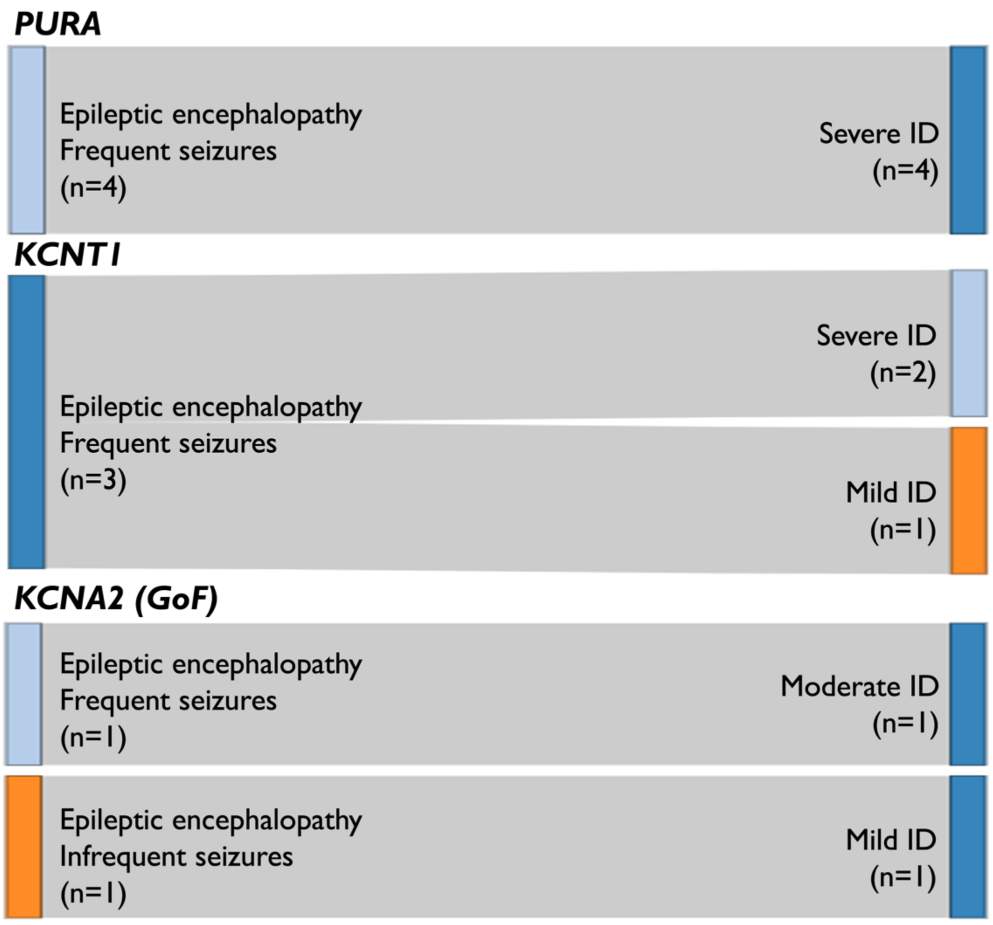

Supplementary Fig. 3. Spectrum of clinical and EEG outcomes for individuals of the non-*SCN1A* cohort for genes with an increasing temporal trend of gene expression. The data are illustrated as Sankey plots to compare with Fig 5, though there are few variations in outcomes to present. Gene-specific DEEs with n>1 individuals are presented. Outcomes presented here were from individuals with complete EEG, seizure and cognitive data in adulthood. Frequent seizures were classified as ≥monthly seizures, infrequent seizures were classified as < monthly seizures. Seizure-free were individuals who have been free of seizures for ≥12 months.

Abbreviations: ID: intellectual disability

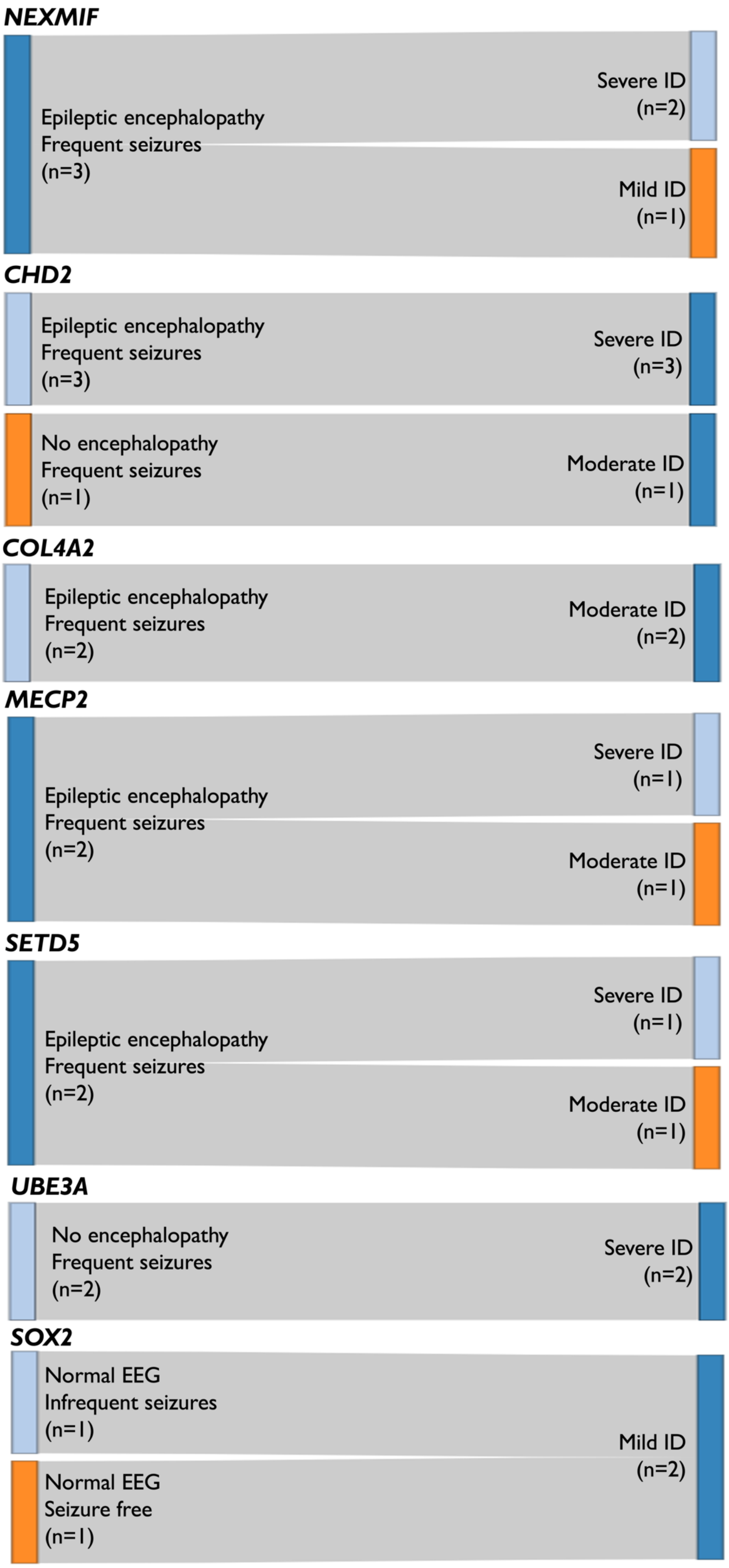
NEXMIF
Epileptic encephalopathy
Frequent seizures
(n=3)
Severe ID
(n=2)
Mild ID
(n=1)
CHD2
Epileptic encephalopathy
Frequent seizures
(n=3)
Severe ID
(n=3)
No encephalopathy
Frequent seizures
(n=1)
Moderate ID
(n=1)
COL4A2
Epileptic encephalopathy
Frequent seizures
(n=2)
Moderate ID
(n=2)
MECP2
Epileptic encephalopathy
Frequent seizures
(n=2)
Severe ID
(n=1)
Moderate ID
(n=1)
SETD5
Epileptic encephalopathy
Frequent seizures
(n=2)
Severe ID
(n=1)
Moderate ID
(n=1)
UBE3A
No encephalopathy
Frequent seizures
(n=2)
Severe ID
(n=2)
SOX2
Normal EEG
Infrequent seizures
(n=1)
Normal EEG
Seizure free
(n=1)
Mild ID
(n=2)

Supplementary Fig. 4. Spectrum of clinical and EEG outcomes for individuals of the non-*SCN1A* cohort for genes with a decreasing temporal trend of gene expression. The data are illustrated as Sankey plots to compare with Fig 5, though there are few variations in outcomes to present. Gene-specific DEEs with n>1 individuals are presented. Outcomes presented here were from individuals with complete EEG, seizure and cognitive data in adulthood. Frequent seizures were classified as ≥monthly seizures, infrequent seizures were classified as < monthly seizures. Seizure-free were individuals who have been free of seizures for ≥12 months.
Abbreviations: ID: intellectual disability

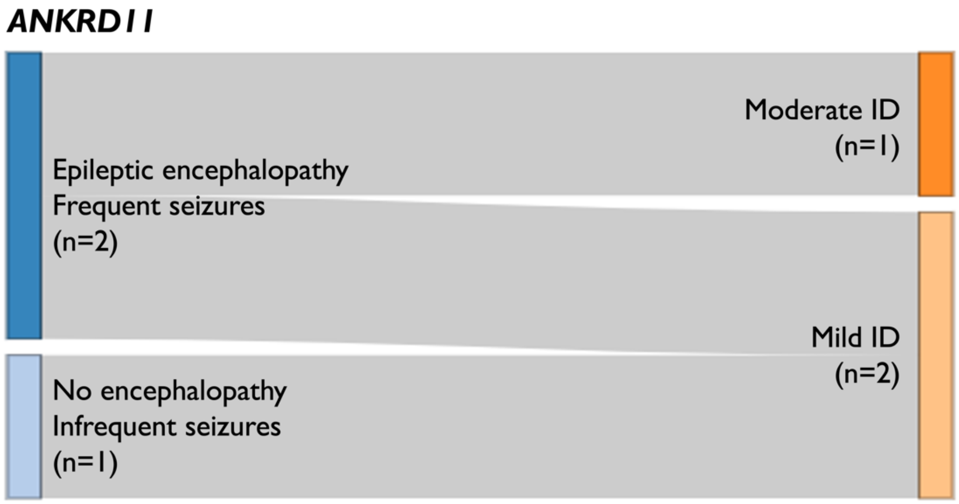

Supplementary Fig. 5. Spectrum of clinical and EEG outcomes for individuals of the non-*SCN1A* cohort for genes with a neutral temporal trend of gene expression. The data are illustrated as Sankey plots to compare with Fig 5, though there are few variations in outcomes to present. Gene-specific DEEs with n>1 individuals are presented. Outcomes presented here were from individuals with complete EEG, seizure and cognitive data in adulthood. Frequent seizures were classified as ≥monthly seizures, infrequent seizures were classified as < monthly seizures. Seizure-free were individuals who have been free of seizures for ≥12 months.
Abbreviations: ID: intellectual disability

3. Supplementary Tables

Supplementary Table I. Linear regression analysis results for age-related gene expression values across the temporal axis. Genes with a positive slope in their regression analysis were considered as having increasing expression trend over time,

whereas genes with a negative slope were considered to have a decreasing expression trend over time. Genes for which the confidence interval of the slope encompassed zero were characterised as having neutral expression over time. The analysis included only the genes implicated in cases for which EEG data were available in adulthood (n=38). Genes are ordered based on their gene expression trend: decreasing, neutral and increasing.

Gene	Slope_LR	CI_2.5	CI_97.5	Temporal expression trend
SOX2	-1.3126849740315	-1.6841323121024	-0.941237635960604	**decreasing**
COL4A2	-0.528139208844416	-0.700816693673802	-0.35546172401503	**decreasing**
COL4A1	-0.521301005346135	-0.699937073458864	-0.342664937233407	**decreasing**
SMS	-0.336559123583198	-0.394609099090169	-0.278509148076228	**decreasing**
UBE3A	-0.33370314100483	-0.389733418381075	-0.277672863628585	**decreasing**
CNTNAP2	-0.321737685812267	-0.475888077273631	-0.167587294350904	**decreasing**
KDM3B	-0.243804600386528	-0.282412525659608	-0.205196675113447	**decreasing**
DNMT3A	-0.227525311727618	-0.254589298159952	-0.200461325295284	**decreasing**
NEXMIF	-0.18925041536596	-0.211442388669844	-0.167058442062077	**decreasing**
PCDH19	-0.149022146244876	-0.191364310937488	-0.106679981552265	**decreasing**
CHD2	-0.135865747374502	-0.152036335751766	-0.119695158997239	**decreasing**
SETD1B	-0.120914762366657	-0.140207551700821	-0.101621973032493	**decreasing**
KCNQ2	-0.110896186832021	-0.167123573850954	-0.0546687998130876	**decreasing**
SETD5	-0.110450138380542	-0.133092646908487	-0.0878076298525975	**decreasing**
ARID2	-0.100275874714295	-0.112480086682814	-0.0880716627457747	**decreasing**
PTEN	-0.0961349021725148	-0.125615010650166	-0.0666547936948634	**decreasing**
AP4M1	-0.0636709701260538	-0.0806305596387743	-0.0467113806133333	**decreasing**
MECP2	-0.0442617416939318	-0.062701930459953	-0.0258215529279106	**decreasing**
GNAO1	0.109208073733894	-0.118480213913272	0.33689636138106	**neutral**
LIS1	0.180864293044426	-5.0769937641193E-06	0.361733663082616	**neutral**
SATB1	-0.0508524221462129	-0.113219073573784	0.0115142292813582	**neutral**
GAMT	-0.00754328788998743	-0.0595457837833381	0.0444592080033633	**neutral**
DEAF1	-0.00667865027572691	-0.133313737982419	0.119956437430965	**neutral**
ANKRD11	0.00901912324376099	-0.0180525344128376	0.0360907809003596	**neutral**
AP4S1	0.034849956956871 1	0.0277456533732971	0.0419542605404451	**increasing**
PURA	0.0970453850261694	0.0840945557912766	0.109996214261062	**increasing**
KCNT1	0.159853880730927	0.140795114481139	0.178912646980715	**increasing**
SCN8A	0.274065009026423	0.23334126626808	0.314788751784766	**increasing**
SCN1A	0.310393792163046	0.276996131327218	0.343791452998874	**increasing**
SCN2A	0.33976205724031	0.266828904392924	0.412695210087695	**increasing**
KCNA2	0.365859646899488	0.331079390076314	0.400639903722662	**increasing**
GABRA1	1.38722224253707	1.23307758666226	1.54136689841188	**increasing**

KIF1A	1.39370573313359	1.11908061563946	1.66833085062772	**increasing**
SLC6A1	1.50132850219684	1.29845358632099	1.70420341807269	**increasing**
DNM1	2.99895902533604	2.68710720950204	3.31081084117003	**increasing**
ATP1A2	3.30113008997417	2.58830473891551	4.01395544103283	**increasing**
STXBP1	3.6305516054444	3.15268184129704	4.10842136959176	**increasing**
CAMK2A	7.57149542858842	6.84966327072043	8.2933275864564	**increasing**

Supplementary Table 2. Median age and age ranges at time of inclusion in the study, age at genetic diagnosis and age at clinical diagnosis of DEE (if made prior to genetic diagnosis), for the *SCN1A* and non-*SCN1A* cohorts. Genes for the non-*SCN1A* cohort are presented in alphabetic order.

Median age (age range)[a], at inclusion in the study	Median age (age range), at genetic diagnosis	Median age (age range), at clinical diagnosis of DEE<br>N: number of individuals, if any, with a clinical diagnosis of DEE prior to genetic diagnosis	Genes (for non-*SCN1A* cohort) (N)[b]
***SCN1A* cohort**			
30-39 (10-79)	26 (5-60)	26 (1-60)<br>N=41	*SCN1A* (59)
**Non-*SCN1A* cohort**			
20-29 (20-49)	27 (17-39)	25 (17-39)<br>N=1	*ANKRD11* (3)
20-29 (20-29)	25 (24-26)	22 (21-23)<br>N=2	*AP4M1* (2)
20-29	24	22<br>N=1	*AP4S1* (1)
20-29	25	23<br>N=1	*ARID2* (1)
20-29	27	25<br>N=1	*ATP1A2* (1)
20-29	29	29	*CAMK2A* (1)
20-29 (10-49)	26.5 (14-42)	20.5 (5-42)<br>N=3	*CHD2* (6)
30-39	25	25	*CNTNAP2* (1)
30-39 (20-49)	23 (17-29)	22(17-27)<br>N=1	*COL4A1* (2)
30-39	31	27<br>N=1	*COL4A2* (1)
10-19	16	16	*DEAF1* (1)
30-39	24	21<br>N=1	*DNM1* (1)
20-29	28	23<br>N=1	*DNMT3A* (1)
20-29	23	23	*GABRA1* (1)
30-39 (30-39)	29.5 (26-33)	25.5 (23-28)<br>N=2	*GAMT* (2)
30-39	31	21	*IQSEC2* (1)

		N=1	
20-29	20	18	*GNAO1* (1)
		N=1	
20-29 (10-49)	23 (19-37)	23 (16-37)	*KCNA2* (3)
		N=1	
30-39 (10-49)	27.5 (13-42)	26.5 (13-40)	*KCNQ2* (2)
		N=1	
30-39 (20-49)	29 (21-43)	27 (17-43)	*KCNT1* (3)
		N=2	
30-39	33	32	*KDM3B* (1)
30-39	26	26	*KDM6A* (1)
40-49 (30-59)	44.5 (38-51)	44.5 (38-51)	*KIF1A* (2)
20-29	22	22	*LIS1* (1)
40-49 (20-49)	40 (25-45)	40 (23-45)	*MECP2* (3)
		N=1	
20-29 (20-59)	28 (24-48)	25 (24-48)	*NEXMIF* (3)
		N=1	
30-39	31	23	*PCDH19* (1)
		N=1	
20-29	21	18	*PTEN* (1)
		N=1	
20-29 (20-49)	25 (17-41)	24.5 (17-41)	*PURA* (4)
		N=1	
20-29	20	13	*SATB1* (1)
20-29 (20-29)	16 (14-21)	16 (14-21)	*SCN2A* (3)
20-29	23	23	*SCN8A* (1)
20-29	22	19	*SETD1B* (1)
30-39 (20-49)	30 (22-38)	30 (22-38)	*SETD5* (2)
40-49 (40-49)	36 (35-37)	35.5 (35-36)	*SLC2A1* (2)
		N=1	
40-49	47	47	*SLC6A1* (1)
20-29	20	17	*SMS* (1)
20-29 (20-39)	27.5 (20-35)	27.5 (20-35)	*SOX2* (2)
10-19	10	10	*STXBP1* (1)
40-49	40	40	*TBC1D24* (1)
30-39 (20-49)	37 (37)	37 (37)	*UBE3A* (2)

[a] Median age and age ranges presented in decadal ranges to minimise risk of identification.

[b] N: total number of individuals

Abbreviations: DEE: developmental and epileptic encephalopathy

Supplementary Table 3. Gene expression trends across different clinical and EEG phenotypes. EE: presence of encephalopathic EEG features in combination with epileptiform activity and ongoing clinical seizures. Encephalopathy: presence of encephalopathic EEG features but without epileptiform activity or with epileptiform activity but without clinical seizures. No Encephalopathy: no features of encephalopathy on EEG but on-going epileptiform activity and/or clinical seizures. Normal EEG: with or without ongoing clinical seizures. Genes in bold are those appearing in more than electroclinical outcome category.

Temporal trend of gene expression	EE; Point prevalence N (%) and genes involved	Encephalopathy; Point prevalence N (%) and genes involved	No Encephalopathy; Point prevalence N (%) and genes involved	Normal EEG; Point prevalence N (%) and genes involved
	Total prevalence: 69/99 (69.7)	Total prevalence: 16/99 (16.1)	Total prevalence: 8/99 (8.1%)	Total prevalence: 6/99 (6.1)
Increasing	42/69 (60.9)	11/16 (68.8)	5/8 (62.5)	3/6 (50)
	SCN1A (26)<br>*AP4S1*<br>*ATP1A2*<br>*DNM1*	***SCN1A*** (9)<br>*CAMK2A*<br>***KCNA2***	***SCN1A*** (3)<br>*GABRA1*<br>*KIF1A*	***SCN1A*** *(3)*

	KCNA2 *(2)*<br>*KCNT1 (3)*<br>*PURA (4)*<br>*SCN2A*<br>*SCN8A*<br>*SLC6A1*<br>*STXBP1*			
Neutral	5/69 (7.2)	2/16 (12.5)	1/8 (12.5)	0
	ANKRD11 *(2)*<br>*DEAF1*<br>*LIS1*<br>*SATB1*	*GAMT*<br>*GNAO1*	***ANKRD11***	0
Decreasing	22/69 (31.9)	3/16 (18.7)	2/8 (25)	3/6 (50)
	AP4M1<br>*ARID2*<br>***CHD2*** *(3)*<br>*COL4A1 (2)*<br>***KCNQ2***<br>*NEXMIF (3)*<br>*MECP2 (2)*<br>*PCDH19*<br>*PTEN*<br>*SETD1B*<br>*SETD5 (2)*<br>*SMS*<br>*UBE3A (2)*	*COL4A2*<br>*DNMT3A*<br>***KCNQ2***	***CHD2***<br>*KDM3B*	*CNTNAP2*<br>*SOX2 (2)*

Abbreviations: EE: epileptic encephalopathy

Supplementary Table 4. Clinical management changes after genetic diagnosis. Information on management changes was available for 124/129 (96.1%) individuals. Changes were implemented for 69/124 (55.6%) individuals, whereas for the remaining 55/124 (45.4%) no changes specific to the genetic diagnosis were made. All the individuals harbouring pathogenic *SCN1A* variants here, had a diagnosis of Dravet syndrome. Genes in bold are those appearing in more than specialist referral category.

Management change	Point prevalence; N (%)	Genes (N)	Details
**ASM change/initiation**	28/124 (22.6)	*SCN1A (25)*	Withdrawal of sodium channel blocking ASMs or introduction of STP
		SCN2A (1)[a]	Trial of sodium channel blocking ASM
		SLC2A1 (1)	Initiation of KD
		KCNA2 (1)	Trial of fampiridine
**Specialist referral**[b]	48/124 (38.7)	***ANKRD11***<br>***COL4A1***<br>***COL4A2***<br>*DNMT3A*<br>*KCNT1*<br>***KDM6A***<br>*MECP2*<br>*SCN2A*<br>*STED1B*<br>***SCN1A***<br>*(20)*	referral for cardiac screening
		ANKRD11<br>***COL4A1***<br>***COL4A2***<br>***KDM6A***<br>*SMS*<br><br>*(6)*	referral for renal screening
		COL4A1<br>***COL4A2***<br>*(2)*	referral for ophthalmological screening
		PTEN<br>*(1)*	referral for cancer screening
		AP4M1<br>*KIF1A*<br>*(4)*	referral to neurology specialist
		SOX2<br>*(1)*	referral for gastrointestinal screening (pancreatic insufficiency screening)
		GAMT<br>*(1)*	referral to metabolic specialist
		AP4M1	referral for family (other than parental) genetic testing

(1)	
SCN1A (12)	referral to learning disability psychiatry specialist
SCN1A (3)	referral to orthopaedic and physiotherapy specialists

[a] The individual was known to harbour a gain-of-function pathogenic variant.
[b] Referrals to specialists are presented by group of genes to minimise risk of identification. 25 individuals with specialist referrals are included in more than one referral categories.

Supplementary Table 5. Temporal progression of clinical and functional domains across the *SCN1A* (n=59) and non-*SCN1A* (n=70) cohorts between childhood and adulthood and relevant genes involved. Genes in bold are those appearing in more than one category. As most data were collected retrospectively from medical records, parameters had rarely been evaluated using formal scales. Changes were considered to be present when reported by families, carers and clinicians as documented in medical records. Genes in bold are those appearing in more than one category. The parentheses in the fourth column indicate the number of individuals involved when greater than one.

**Progression**	***SCN1A* cohort; Number of individuals (%)**	**Non-*SCN1A* cohort; Number of individuals (%)**	**Genes (for non-*SCN1A* cohort)**
**Seizures**			
Improvement	33/59 (55.9)	21/70(30)	***AP4M1***, *AP4S1*, *CAMK2A*, ***CHD2,*** *COL4A2*, *DEAF1*, *DNMT3A*, ***GAMT,*** *KCNA2* (3), ***KCNQ2***, *KDM3B*, *KDM6A*, *PTEN*, ***SCN2A*** (2), *SLC6A1*, ***SOX2***, *STXBP1*, ***UBE3A***
No difference	17/59 (28.8)	28/70 (40)	*ANKRD11* (3), ***AP4M1***, *ARID2*, *ATP1A2*, ***CHD2*** (2), *CNTNAP2*, ***COL4A1,*** *IQSEC2*, *GABRA1*, ***GAMT, KCNT1***(2), *KIF1A* (2), *PCDH19*, ***PURA*** (2), *SCN8A*, *SETD1B*, *SETD5* (2), *SLC2A* (2), ***SOX2***, *TBC1D24*
Deterioration	9/59 (15.3)	21/70(30)	***CHD2*** (3), ***COL4A1***, *DNM1*, *GNAO1*, ***KCNQ2, KCNT1,*** *NEXMIF* (3), *LIS1*, *MECP2* (3), ***PURA*** (2), *SATB1*, ***SCN2A***, *SMS*, ***UBE3A***
**Cognition/Language**			
Improvement	6/59 (10.2)	1/70 (1.4)	*CAMK2A*
No difference	45/59 (76.3)	64/70 (91.5)	*ANKRD11* (3), *AP4M1* (2), *AP4S1*, *ARID2*, *ATP1A2*, ***CHD2*** (5), *CNTNAP2*, *COL4A1* (2), *DEAF1*, *DNM1*, *IQSEC2*, *GABRA1*, *GAMT* (2), *GNAO1* , *KCNA2* (3), ***KCNQ2***, *KCNT1* (3), *KDM3B*, *KDM6A*, *NEXMIF* (3), *KIF1A* (2), *LIS1*, ***MECP2*** (2), *PCDH19*, *PTEN*, *PURA* (4), *SATB1*, *SCN2A* (3), *SCN8A*, *SETD1B*, *SETD5* (2), *SLC2A1* (2), *SLC6A1*, *SMS*, ***SOX2***, *STXBP1*, *TBC1D24*, *UBE3A* (2),
Deterioration	8/59 (13.5)	5/70 (7.1)	***CHD2***, *DNMT3A*, ***KCNQ2***, ***MECP2***, ***SOX2***
**Behaviour**[a]			
Improvement	0	0	-
No difference	37/42 (88.1)	29/33 (87.9)	*ANKRD11* (3), *CAMK2A*, *CHD2* (3), *CNTNAP2*, *COL4A1* (2), *COL4A2*, *DEAF1*, *DNMT3A*, *GAMT* (2), *KCNT1* (2), *KDM6A*, *NEXMIF* (2), *KIF1A* (2), *PCDH19*, *PTEN*, *SCN2A*, *SETD1B*, *SETD5*, *SLC2A1*, *SMS*
Deterioration	5/42 (11.9)	4/33 (12.1)	*KCNQ2* (2), *KDM3B*, *SOX2*
**Mobility**[a]			
Improvement	0	0	-
No difference	4/37 (10.8)	13/29 (44.8)	***AP4M1***, *AP4S1*, *CHD2*, *DNM1*, *IQSEC2*, *KCNQ2* (2), *LIS1*, ***PURA*** (2), *SCN2A*, *SLC2A1*, *SLC6A1*

Deterioration	33/37 (89.2)	16/29 (55.2)	***AP4M1***, *GNAO1*, *KCNA2* (2), *NEXMIF*, *KIF1A*, *MECP2*, *PURA* (2), *SATB1*, *SETD5*, *SMS*, *STXBP1*, *TBC1D24*
**Feeding**[a]			
Improvement	0	0	-
No difference	0	3/12 (25)	*KDM3B*, ***PURA***, *SETD5*
Deterioration	20/20 (100)	9/12 (75)	*CHD2*, *KCNQ2*, *NEXMIF*, *LIS1*, *MECP2*, ***PURA***, *SCN2A* (2), *TBC1D24*

[a] Denominators in these domains represent the number of individuals with reported difficulties for each specific domain.

Supplementary Table 6 Comparison of childhood and adulthood EEG data for the individuals with available data (n=24).

**Case ID**	***Gene***	**(Age[a]) EEG in childhood**	**(Age[a]) EEG in adulthood**
1-1.54	*SCN1A*<br>Dravet syndrome	0-9 years:<br>Encephalopathy<br>Photosensitivity.<br>IEDs left-sided	10-19 years:<br>Encephalopathy<br>Bilateral IEDs
1-1.57	*SCN1A*<br>Dravet syndrome	0-9 years:<br>Encephalopathy<br>Multifocal IEDs<br>Generalised IEDs during photic stimulation	20-29 years:<br>Encephalopathy<br>Multifocal IEDs<br>Focal-onset seizures
1-1.65	*SCN1A*<br>Dravet syndrome	10-19 years:<br>Encephalopathy<br>rhythms<br>Multifocal IEDs	20-29 years:<br>Encephalopathy<br>Multifocal IEDs<br>Focal-onset seizures
1-1.70	*SCN1A*<br>Dravet syndrome	10-19 years:<br>Formed background rhythms<br>Multifocal IEDs<br>Multifocal seizures	10-19 years:<br>Formed background rhythms<br>Multifocal IEDs<br>Generalised seizures
1-1.72	*SCN1A*<br>Dravet syndrome	10-19 years:<br>Encephalopathy<br>Multifocal IEDs<br>Focal seizures	30-39 years:<br>Encephalopathy<br>Bilateral IEDs
1-1.87	*SCN1A*<br>Dravet syndrome	10-19 years:<br>Encephalopathy<br>Multifocal IEDs<br>Myoclonic status	20-29 years:<br>Encephalopathy<br>Multifocal IEDs<br>Myoclonic seizures<br>Generalised seizures
1-1.89	*SCN1A*<br>*SCN1A*-related epilepsy	0-9 years:<br>Within normal limits	20-29 years:<br>Encephalopathy<br>Bilateral/Focal IEDs
1-1.94	*SCN1A*<br>Dravet syndrome	0-9 years:<br>Within normal limits	30-39 years:<br>Encephalopathy<br>Bilateral/Multifocal IEDs<br>Focal seizures
1-1.96	*SCN1A*<br>Dravet syndrome	0-9 years:<br>Encephalopathy<br>Generalised IEDs	20-29 years:<br>Encephalopathy<br>Multifocal IEDs<br>Focal-onset seizures
1-1.97	*SCN1A*<br>Dravet syndrome	10-19 years:<br>Encephalopathy<br>Bilateral IEDs	30-39 years:<br>Encephalopathy<br>Multifocal IEDs<br>Focal seizures
1-1.99	*SCN1A*<br>Dravet syndrome	10-19 years:<br>Encephalopathy<br>Bilateral IEDs<br>Focal seizures	20-29 years:<br>Encephalopathy<br>Focal IEDs<br>Focal seizures
1-1.104	*SCN1A*<br>Dravet syndrome	0-9 years:<br>Within normal limits	20-29 years:<br>Within normal limits
1-1.29	*KCNA2*	10-19 years:	10-19 years:

		Encephalopathy<br>Focal/Generalised IEDs	Encephalopathy<br>No epileptiform activity
1-1.2	*ANKRD11*	10-19 years:<br>Within normal limits	20-29 years:<br>No encephalopathy (formed background rhythms)<br>Focal IEDs
1-1.4	*AP4M1*	0-9 year:<br>Hypsarrhythmia	10-19 years:<br>Encephalopathy<br>Multifocal IEDs
1-1.17	*COL4A1*	0-9 years:<br>Encephalopathy<br>Bilateral IEDs	10-19 years:<br>Encephalopathy<br>Bilateral IEDs
1-1.20	*DNM1*	0-9 years:<br>Lennox-Gastaut syndrome	20-29 years:<br>Encephalopathy<br>Multifocal IEDs
1-1.21	*DNMT3A*	10-19 years:<br>Encephalopathy<br>No epileptiform activity	20-29 years:<br>Encephalopathy<br>No epileptiform activity
1-1.42	*LIS2*	0-9 years:<br>No details on background rhythms<br>No epileptiform activity	20-29 years:<br>Lennox-Gastaut syndrome
1-1.50	*PURA*	10-19 years:<br>Epileptic Encephalopathy<br>Continuous IEDs	10-19 years:<br>Epileptic Encephalopathy<br>Very frequent IEDs and focal seizures
1-1.115	*SETD1B*	10-19 years:<br>Encephalopathy<br>Focal IEDs<br>Generalised seizures	20-29 years:<br>Encephalopathy<br>Generalised IEDs<br>Generalised seizures
1-1.116	*SETD5*	10-19 years:<br>Encephalopathy<br>Focal IEDs	20-29 years:<br>Encephalopathy<br>Focal IEDs
1-1.122	*SOX2*	0-9:<br>Encephalopathy<br>No epileptiform activity	30-39 years:<br>Within normal limits
1-1.124	*STXBP1*	0-9 year:<br>Within normal limits	10-19 years:<br>Encephalopathy<br>Focal seizures

[a] Age at time of the EEG study is presented in decadal range to minimise risk of identification.
